# Single‐cell imaging of genome organization and dynamics

**DOI:** 10.15252/msb.20209653

**Published:** 2021-07-07

**Authors:** Liangqi Xie, Zhe Liu

**Affiliations:** ^1^ Janelia Research Campus Howard Hughes Medical Institute Ashburn VA USA

**Keywords:** chromatin dynamics, genome organization, imaging, single cell, super‐resolution, Chromatin, Epigenetics, Genomics & Functional Genomics

## Abstract

Probing the architecture, mechanism, and dynamics of genome folding is fundamental to our understanding of genome function in homeostasis and disease. Most chromosome conformation capture studies dissect the genome architecture with population‐ and time‐averaged snapshots and thus have limited capabilities to reveal 3D nuclear organization and dynamics at the single‐cell level. Here, we discuss emerging imaging techniques ranging from light microscopy to electron microscopy that enable investigation of genome folding and dynamics at high spatial and temporal resolution. Results from these studies complement genomic data, unveiling principles underlying the spatial arrangement of the genome and its potential functional links to diverse biological activities in the nucleus.

## Graphical abstract

Imaging technologies have been developed rapidly in recent years. This Review discusses emerging single‐cell imaging techniques and their applications in investigating genome folding and dynamics at high spatial and temporal resolution.

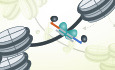

## Introduction

The eukaryotic cell nucleus is a complex biological system that hosts the genomic DNA in the form of chromatin. A myriad of regulatory factors (proteins, RNAs, metabolites, etc.) reside in the nucleus and participate in biological activities that decode, transmit, and maintain the genetic information (*i.e*., transcription, DNA replication, DNA repair).

**Figure 1:**
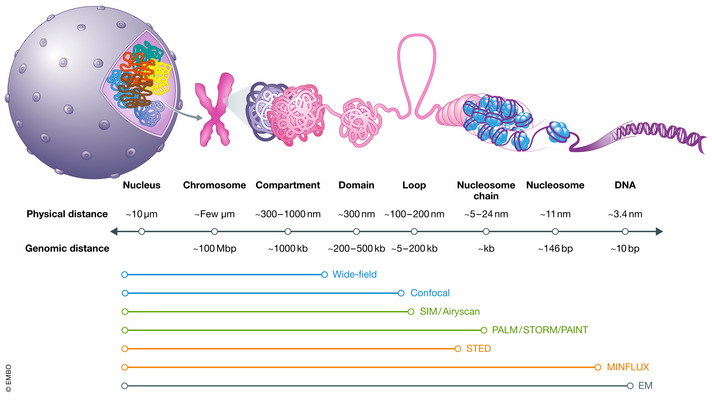
Spatial scale of genome organization The mammalian genome is hierarchically organized at distinct spatial scales. From right to left, DNA is wrapped onto histone octamers and forms a chain of nucleosome clutches. The chromatin fiber folds into chromatin loops which are further organized into domains (e.g., TADs). Domains with similar chromatin state and activity are coiled together into compartments (e.g., active A and inactive B) across individual chromosomes (for simplicity, one mitotic chromosome is shown). The physical scale and genomic distance are indicated as below, along with microscopy methods suitable for probing the corresponding scale.

How the human genome with a total DNA length of ~2 m is wrapped onto nucleosomes and further folded into chromosomes remains a mystery in cell biology. In past decades, genome organization has been extensively probed by genomic methods based on chromatin conformation capture (3C) and its variants (*e.g*.,4C, 5C, Hi‐C, Micro‐C; Dekker *et al*, 2002; de Wit & de Laat, 2012; Hsieh *et al*, 2015; Dekker & Mirny, 2016). In 3C‐based assays, the “distance” between two genomic positions is estimated by their contact frequency or the proximity‐ligation probability after chemical crosslinking and nuclease fragmentation. One unique advantage of 3C‐based techniques is that the sequence information is inherently embedded in the data and millions of pairwise contact frequencies are measured in parallel. As a result, these techniques have provided significant insights into chromosome folding at a wide range of length scales (Fig 1). Specifically, it was found that chromosomes are organized into mega‐base pair (Mbp)‐sized active (A) and inactive (B) compartments that are further folded into sub‐Mbp topologically associating domains (TADs) and then into even smaller contact loops (Lieberman‐Aiden *et al*, 2009; Dixon *et al*, 2012; Nora *et al*, 2012; Sexton *et al*, 2012; Rao *et al*, 2014). Compartments correlate well with chromatin state‐specific epigenetic marks, and TADs appear to constrain chromatin interactions (*e.g*., enhancer‐promoter) within their boundaries marked by convergent CTCF sites. Contact loops are thought to form by a loop extrusion mechanism driven by the Cohesin ring (Alipour & Marko, 2012; Sanborn *et al*, 2015; Fudenberg *et al*, 2016). Recently, protein‐mediated chromatin contact maps have been generated by combining chromatin immunoprecipitation with proximity ligation (Tang *et al*, 2015; Mumbach *et al*, 2016). We here refer to a few excellent reviews that comprehensively cover the concepts, methods, and insights from these genomics studies (Dekker & Mirny, 2016; Yu & Ren, 2017; Rowley & Corces, 2018; Kempfer & Pombo, 2020; McCord *et al*, 2020).

The pairwise contact frequency measured by 3C‐based methods, however, does not always correlate with the physical distance of loci pairs (Williamson *et al*, 2014), raising the intriguing possibility that the nonlinearity associated with chemical crosslinking, variation of proximity‐ligation efficiency in heterogeneous chromatin environments, and non‐equilibrium loop extrusion dynamics could hinder faithful reconstruction of the 3D spatial genome architecture from genomic data (Fudenberg & Imakaev, 2017). In addition, recent single‐cell Hi‐C assays revealed substantial variability of chromatin structures in individual cells and found that TADs represent reconstitution after population averaging (Bintu *et al*, 2018), suggesting that 3C‐based techniques mainly capture cell population‐ and time‐averaged “snapshots” of the genome configuration. It is worth mentioning that several ligation‐free genomic methods have been developed to map higher‐order chromatin interactions and positioning relative to nuclear landmarks (e.g., nuclear envelope and bodies) by sequencing thin cryo‐sectioned nuclei (Beagrie *et al*, 2017; preprint: Fiorillo *et al*, 2020), DNA‐chromatin complexes (Quinodoz *et al*, 2018; Zheng *et al*, 2019), and proximity‐based enzymatic modifications (van Steensel & Belmont, 2017; Chen *et al*, 2018b; Girelli *et al*, 2020). However, these methods still involve sectioning or destruction of cells and rely on statistical models to indirectly infer chromatin arrangement in the nucleus.

To address these limitations, emerging microscopy approaches have been developed to directly observe genomic features in fixed samples and track chromatin dynamics in live cells. Results from these studies begin to piece together a single‐cell view of the 3D genome topology complementary to what is derived from genomic data, revealing new insights underlying genome organization and function.

## Imaging the spatial organization of the genome

Light microscopy (LM) and electron microscopy (EM) are indispensable tools for studying genome folding which occurs at a wide range of length scales spanning several orders of magnitude (Fig 1). No one‐size‐fit‐all microscope exists to capture all scales at once. LM revealed that chromosomes (up to ~100 Mbp) form ~micron‐sized “territories” (Cremer & Cremer, 2001, 2010) but is limited at resolving smaller genomic features, due to the Abbe diffraction limit (~200–300 nm in *xy* and ~600 nm in *z*). EM can image molecular structures (e.g., nucleosomes ~10 nm in diameter) down to the atomic level. However, chromatin fibers generally have low contrast in commonly used EM stains. Therefore, a knowledge gap exists between 10 nm and 200 nm (~150 bp to a few hundred kilobase pairs or kbp). Within this range, essential biological activities occur, such as chromatin looping, nucleosome packing, enhancer‐promoter interactions, and the assembly of transcription and replication machineries.

Recent development of super‐resolution imaging techniques and labeling tools has broken barriers in both LM and EM at unprecedented resolution and scale. Here, we provide an overview of these technologies and conceptual advances derived from them (Table 1).

**Table 1:** List of major imaging methods to study 3D genome organization and dynamics.

Methods	Description	Throughput	Microscope/Resolution	Measurements	References
Fluorescence Light Microscopy					
3D FISH	BAC/fosmid/ PCR‐derived double‐stranded probe for in situ hybridization	Tens to hundreds of cells	Confocal	Gene positioning, 3D distance	Solovei and Cremer (2010), Bienko *et al* (2013)
HIPMap	High‐throughput FISH, automatic image and statistical analysis	Hundreds of cells	Opera confocal high‐throughput imaging system	Gene positioning, 3D distance	Shachar *et al* (2015)
CryoFISH	Cryosectioning + FISH in 2D	Tens to hundreds of cells	Confocal	High‐resolution 2D‐distance	Branco and Pombo (2006), Barbieri *et al* (2017)
OligoSTORM	Oligopaint(single strand probe)+STORM; large step size (30 kbp‐1Mbp)	Tens to hundreds of cells	STORM, super‐resolution	Volume, density, surface area, domain overlap	Beliveau *et al* (2015), Wang *et al* (2016), Bintu *et al* (2018), Nir *et al* (2018)
OligoDNA‐PAINT	Oligopaint +DNA‐PAINT	Tens of cells	DNA‐PAINT, super‐resolution	Nanoscale domain structure	Beliveau *et al* (2015)
ORCA	Oligopaint+barcoding+sequential imaging (2‐10 kbp bin)	Thousands of cells	Wild field + Auto‐fluidics	Chromatin folding path tracing	Mateo *et al* (2019)
Hi‐M	Oligopaint + barcoding + sequential imaging (~4 kbp bin)	Thousands of cells	Wild field + Auto‐fluidics	Chromatin folding path tracing	Cardozo Gizzi *et al* (2019)
DNA‐MERFISH	Oligopaint + combinatorial barcoding and decoding	Thousands of cells	Custom built microscope + Auto‐fluidics	Genome‐scale chromatin organization	Su *et al* (2020)
DNA SeqFISH+	Oligopaint + combinatorial barcoding and decoding	Thousands of cells	Spinning disk confocal+ Auto‐fluidics	Genome‐scale imaging (~1Mb) and chromatin folding (~25kb)	Takei *et al* (2021)
ATAC‐see	Transposase‐assisted integration of fluorescent probes into accessible chromatin	Tens to hundreds of cells	Confocal	Gross accessible chromatin pattern in different cell types and during cell cycle	Chen *et al* (2016)
ATAC‐PALM	Transposase‐assisted integration of photoactivatable fluorescent probes + LLSM_based PALM imaging	Tens of cells	3D whole nucleus super‐resolution (xy ~ 20 nm, z ~ 50 nm)	Accessible chromatin domains architecture (e.g., size, shape, density, connectivity).	Xie *et al* (2020)
3D‐SIM	Structured illumination pattern, Moiré fringes	Tens to hundreds of cells	~2‐fold resolution improvement (xy ~100 nm )	Chromatin and nucleus organization	Schermelleh *et al* (2008), Miron *et al* (2020)
STED	Donut‐shaped beam to deplete fluorescence except at the donut center	Tens of cells	Point scanning, xy ~ 50 nm	Genome organization protein, chromatin regulator	Gu *et al* (2020)
MINFLUX	Stochastic switching, donut‐shaped beam excites fluorescence except at the donut center	Tens of cells	Molecular resolution (xy ~ nm)	Nanometer scale ultrastructure	Balzarotti *et al* (2017)
Electron Microscopy					
ChromEMT	DRAQ5 binds and photosensitizes chromatin DNA followed by SEM and tomography	Tens of cells	SEM and tomography	Ultrastructure of chromosome in situ, 5‐24 nm nucleosome chains	Ou *et al* (2017)
FIB‐SEM	Iterative surface milling by gallium ions + SEM	Tens of cells	SEM ~4‐8 nm xyz	Cellular ultrastructure. Chromatin domains	Miron *et al* (2020)
3D‐EMISH	Cryosectioning + FISH + EM + 3D reconstruction	Hundreds of cells	Scanning EM Improved z	1.7 Mbp chromatin folding	Trzaskoma *et al* (2020)
Correlative Light and Electron Microscopy					
Correlative Cryo‐SR/ FIB‐SEM	Cryo‐fixation, SR light microscopy followed by correlative FIB‐SEM imaging	Tens of cells	Cryogenic SIM/PALM, FIB‐SEM (~4‐8 nm xyz)	Euchromatin and heterochromatin in different cell types	Hoffman *et al* (2020)
Live cell imaging					
SMT	Sparse labeling with self‐labeling tag and tracking in real time	Tens of cells	HILO or TIRF, single‐molecule resolution	Chromatin regulator diffusion, target search and binding dynamics	Mazza *et al* (2012), Gebhardt *et al* (2013), Chen *et al* (2014b), Xie *et al* (2017), Hansen *et al* (2017)
Chromatin tracking	Non‐editing(CRISPR/Cas9) or editing (DNA arrays)	Tens to hundreds of cells	Spinning disk, Airyscan FAST, SIM, LLSM	Chromatin mobility and interactions	Chen *et al* (2013), Ochiai *et al* (2015), Chen *et al* (2018a), Alexander *et al* (2019)

3D‐EMISH, serial block‐face scanning electron microscopy with in situ hybridization; 3D‐SIM, 3D structured illumination microscopy; ATAC‐PALM, assay of transposase‐accessible chromatin with photoactivated localization microscopy; ATAC‐see, assay of transposase‐accessible chromatin with visualization; ChromEMT, chromosome EM tomography; Cryo‐SR, cryo super‐resolution microscopy; DNA SeqFISH+, DNA sequential FISH; DNA‐PAINT, DNA‐based point accumulation for imaging in nanoscale topography; FIB‐SEM, focused ion beam‐scanning electron microscopy; Hi‐M, high‐throughput, high‐resolution, high‐coverage microscopy; HIPMap, high‐throughput imaging positioning mapping; LLSM, lattice light‐sheet microscope; MERFISH, multiplexed error‐robust FISH; MINFLUX, minimal emission fluxes; ORCA, optical reconstruction of chromatin architecture; SMT, single‐molecule tracking; STED, STimulated Emission Depletion Microscopy; STORM, Stochastic Optical Reconstruction Microscopy.

## Fluorescence LM

The discovery of genetically encoded fluorescent proteins, self‐labeling tags, better affinity reagents, and organic dyes has made fluorescence LM a useful tool for biology, owing to the possibility of non‐invasive live imaging and the labeling specificity (Liu *et al*, 2015). Specific genomic DNA labeling can be achieved by DNA fluorescence in situ hybridization (FISH), which hybridizes sequence‐specific, fluorescently labeled DNA probes to the genome in chemically fixed cells. DNA FISH measures the 3D distance between genomic loci and DNA positioning relative to nuclear landmarks.

Recently, a high‐throughput DNA FISH platform (HIPMap) was developed to study gene positioning in single cells at scale. It was found that the chromosome positioning is regulated by nuclear structure components (e.g., nuclear envelope, centromeres), chromatin remodelers, and the DNA replication machinery (Shachar *et al*, 2015) and that the spatial genome organization is highly heterogeneous in single cells, with low chances of co‐localization (<30% for 350 nm cutoff) between two “interacting” loci detected by Hi‐C (Finn *et al*, 2019). These results are consistent with previous single‐cell Hi‐C results that genome folding is intrinsically variable at both the loop and the TAD level (Stevens *et al*, 2017). For better structure preservation, DNA FISH was combined with Tokuyasu cryosectioning (CryoFISH) to image the HoxB gene loci (~700 kb) and revealed homotypic contacts associated with active and poised chromatin states (Barbieri *et al*, 2017). CryoFISH also detected extensive chromosomal intermingling (Branco & Pombo, 2006; Simonis *et al*, 2006), suggesting abundant trans‐contacts between chromosome “territories”, consistent with results from other genomic studies (Loviglio *et al*, 2017; Tan *et al*, 2018; Monahan *et al*, 2019).

Another advancement in DNA FISH technology is the invention of a new type of DNA probes. Specifically, conventional DNA FISH probes are usually long double‐stranded genomic fragments derived from BAC or PCR (tens to hundreds of kbp; Solovei & Cremer, 2010; Bienko *et al*, 2013) with limited abilities to resolve fine genomic features such as enhancers, insulators, and promoters (typically ~200 bp in size). Recent probe designs (named Oligopaints) combine synthetic chemistry and molecular biology to produce massive pools of short, thermodynamically tuned single‐stranded oligos. This technique enables labeling of chromosomal segments at a flexible length scale (~5 kb to a few Mbp) under tunable hybridization conditions and even painting of homologous chromosomes based on single nucleotide polymorphism (Beliveau *et al*, 2012, 2015). High‐density labeling by Oligopaint probes satisfies the Nyquist sampling criteria for reconstruction of chromatin structure beyond the diffraction limit. Multiplexed sequential Oligopaints have also been developed for imaging TAD structures across multiple chromosomes in single cells. The mean spatial distance between TADs was found to correlate well with Hi‐C contact frequencies, cross‐validating ensemble Hi‐C results (Wang *et al*, 2016).

Although laser scanning confocal microscope and its variants have achieved increased spatial resolution, the improvement is generally modest (Huff, 2015). Recently, super‐resolution (SR) imaging‐based techniques have been developed to study genome organization in single cells beyond the diffraction limit. SR microscopy can be broadly classified into single‐molecule localization‐based microscopy (SMLM, including PALM/STORM, etc.), structured illumination microscopy (SIM), and stimulated emission depletion microscopy (STED) (Liu *et al*, 2015).

## Imaging genome organization by SMLM

With the discovery of photoactivatable and photoswitchable fluorophores (Patterson, 2002; Bates *et al*, 2005), SMLM was developed to achieve a resolution far below the diffraction limit (Betzig, 1995; Betzig *et al*, 2006; Hess *et al*, 2006; Rust *et al*, 2006). The ability of SMLM to improve spatial resolution (~20 nm in xy and ~50 nm in z) relies on two key steps—sparse isolation and centroid localization of single molecules in densely labeled samples. The underlying structure is reconstructed based on localization precision and localization density distribution in space (Legant *et al*, 2016). Here, we review recent advances in investigating 3D genome organization by SMLM‐based techniques.

## OligoSTORM and its variants

Stochastic optical reconstruction microscopy (STORM) utilizes stochastic activation of photoswitchable fluorophores to achieve single‐molecule isolation and localization (Rust *et al*, 2006; Bates *et al*, 2007). Such fluorophores are usually attached to affinity reagents (primary or secondary antibodies) for labeling. STORM imaging of histone H2B revealed that nucleosomes are assembled into heterogeneous “clutches” (tens to hundreds of nanometers; Ricci *et al*, 2015). Interestingly, a lower clutch density was found in embryonic stem cells (ESCs) compared with differentiated cells, consistent with the enrichment of 10‐nm accessible chromatin mesh in ESCs revealed by electron spectroscopic imaging (Fussner *et al*, 2010).

Photoswitchable fluorophores can also be conjugated to barcoded Oligopaints probes for STORM (named OligoSTORM), which enables investigation of the genome with ~20–50 nm resolution (Beliveau *et al*, 2015), improving distance measurements, and quantification of domain volume, density, sphericity, overlaps, etc. (Boettiger & Murphy, 2020).

OligoSTORM was used to image chromatin fragments (~10–500 kbp) with distinct epigenetic states (active, inactive, repressed) in Drosophila (Boettiger *et al*, 2016). Consistent with earlier biochemical and conventional FISH measurements (Gilbert *et al*, 2004), active chromatin de‐condenses the most, which may facilitate access by regulatory factors. In contrast, repressed chromatin has the highest degree of compaction and displays minimal overlaps with neighboring active domains, ensuring limited access and robust gene suppression. Similar observations were made by OligoSTORM imaging of 8 Mbp of human chromosome at 100‐1000 kbp steps (Nir *et al*, 2018). OligoSTORM was also used to trace ~1–2 Mbp chromosomal segments in human cells with ~30 kbp bin steps (Bintu *et al*, 2018), showing that chromatin fiber is organized into globular domains, termed TAD‐like domains, with diameters of hundreds of nanometers, consistent with results in Drosophila and mouse cells (Szabo *et al*, 2018, 2020). It was also found that the boundary location between TAD‐like domains is variable with higher probabilities at some but not all CTCF‐binding sites, suggesting that CTCF‐defined TAD boundaries in Hi‐C genomic studies likely result from population averaging. Perhaps, the most surprising result is that disruption of loop extrusion by acute Cohesin depletion does not alter the prevalence of TAD‐like domain structures and only decreases the positioning probability of domain boundaries, suggesting that Cohesin‐independent mechanism(s) must be at play to organize such structures (Bintu *et al*, 2018).

**Figure 2:**
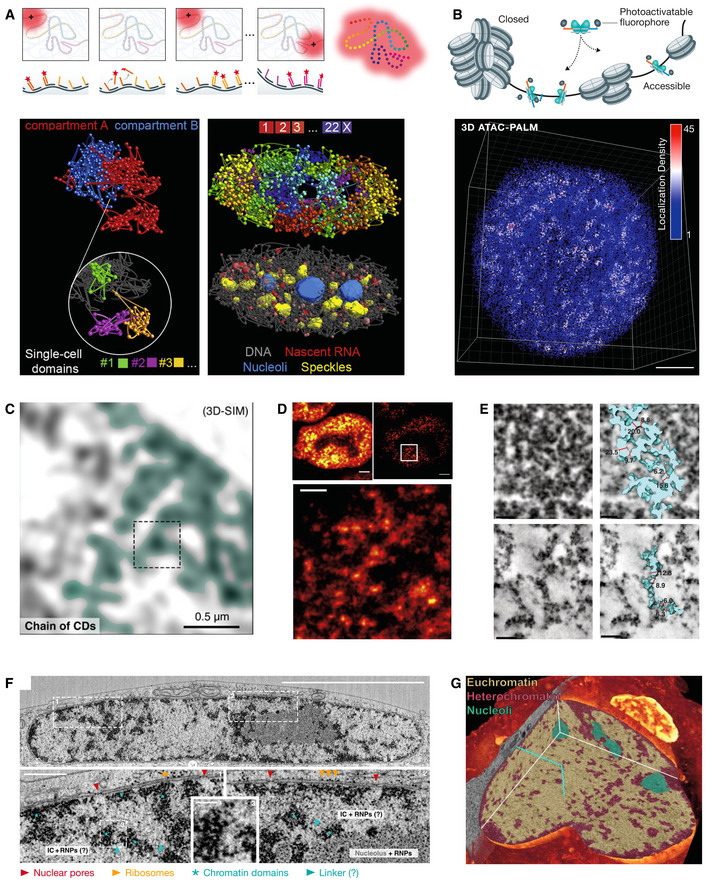
Genome organization revealed by super‐resolution and electron microscopy (A) Probing chromatin folding by Oligopaint DNA FISH. (Upper panel) schematics of the sequential hybridization approach in ORCA. The genomic targets are binned into small DNA segments hybridized with Oligopaint probes carrying distinct barcodes. Each barcode is sequentially bound by complementary fluorescent readout probes, imaged, and enzymatically removed. The centroids of binned DNA segments are used to reconstruct the chromatin structure. Panel adopted from Mateo *et al* (2019) with permission. (Lower left panel) The above process is extended to image multi‐Mbp compartments at smaller bins (lower left) and entire chromosome at larger bins (lower right panel). Panel adopted from Su *et al* (2020) with permission. (B) Imaging the accessible genome architecture by 3D ATAC‐PALM. (Upper panel). The Tn5 transposon conjugated with a photoactivable fluorophore is covalently inserted into accessible genomic sites. (Lower panel) The final 3D reconstructed accessible genome conformation in a single ESC is shown. (C) 3D‐SIM imaging of chromatin labeled with DAPI reveals curvilinear chains of chromatin domains (CDs). Adopted from Miron *et al* (2020) with permission. (D) CTCF with the dL5 peptide that binds the fluorogen malachite green in mouse ESCs. Upper left, a confocal image. Upper right, a STED image of the same cell (lateral resolution ~65 nm). Lower panel, a zoom‐in view of the STED imaging. Panel adopted from Gu *et al* (2020) with permission. (E) ChromEMT reveals 5–24 nm nucleosome chains of various density in the nucleus. Upper panels, mitotic chromosome. Lower panels, interphase chromosome. The left panel shows a single tomographic slice. The right panel shows the rendered chromatin chains. Image adopted from Ou *et al* (2017) with permission. (F) (Upper panel) FIB‐SEM imaging of a cryo‐preserved HeLa cell at 4‐nm isotropic resolution. Scale bar, 5 μm. (Lower panel) Zoom‐in view of two boxed regions. Blue asterisks show 200–300 nm nucleosome clusters with characteristic ~10‐nm‐sized dots. Putative linker segments are shown in blue arrowheads. Adopted from Miron *et al* (2020) with permission. (G) Correlative Cryo‐SIM and FIB‐SEM imaging of the nucleus in the granule neuron progenitor with euchromatin (H3.3) and heterochromatin (HP1) color‐coded. Adopted from Hoffman *et al* (2020) with permission.

Optical reconstruction of chromatin architecture (ORCA) and Hi‐M (high‐throughput, high‐resolution, high‐coverage microscopy) utilize much smaller Oligopaint probe segments (~2–10 kbp) and sequential centroid localization to image fine‐scale chromatin folding (Cardozo Gizzi *et al*, 2019, 2020; Mateo *et al*, 2019). ORCA was multiplexed with RNA FISH to probe the relationship between chromatin folding and gene transcription within a 700 kbp bithorax complex (BX‐C) region at 10‐kbp resolution and a 130‐kbp sub‐segment at 2‐kbp resolution in Drosophila embryos (Mateo *et al*, 2019) (Fig 2A, lower left panel). These high‐resolution and high‐throughput assays (hundreds to thousands of cells) permit investigation of the long‐lasting question regarding enhancer–promoter communication (~ tens of kbp) in single cells even within the tissue context. Surprisingly, it was found that the proximity of BX‐C gene promoter to well‐known enhancers only shows weak association with transcription, different from another report showing that stable enhancer–promoter contacts are coupled with gene activation (Chen *et al*, 2018a).

The enormous barcoding capacity of Oligopaints enables high‐resolution chromatin imaging at scale. Recently, massive multiplexed Oligopaints integrate RNA FISH and protein immunofluorescence to map genome‐scale chromatin organization and transcriptome at high spatial resolution (Su *et al*, 2020). The results validated the previously reported association of transcription states with A/B compartments and nuclear landmarks (Fig 2A, lower right panel). Interestingly, it was found that trans‐chromosomal and long‐range (>75 Mbp) cis‐chromosomal interactions occur preferentially between active compartments, suggesting that regulations specific to active chromatin mediate these contacts. Recently, another multimodal imaging study (DNA SeqFISH+, RNA SeqFISH, and antibody oligo conjugation) interrogated chromatin structure, chromatin states, nuclear bodies, and gene expression in single cells (Takei *et al*, 2021), revealing that combinations of epigenetic marks could define nuclear zones with active genes pre‐positioned near their surfaces.

Taken together, Oligopaint‐based imaging methods, OligoSTORM and also DNA‐PAINT (Jungmann *et al*, 2010; Beliveau *et al*, 2015), when combined with the automated microfluidic system for sequential rounds of hybridization, allow in situ “spatial genomics” study of the structure–function relationship between genome organization and gene regulation. Remarkably, the pairwise distances measured by Oligopaints appear to agree well with the contact frequency matrix from population‐based Hi‐C methods (Wang *et al*, 2016; Bintu *et al*, 2018; Cardozo Gizzi *et al*, 2019; Mateo *et al*, 2019), although the peak Pearson correlation coefficient was found at a relatively large genomic distance (~400–600 nm) (Su *et al*, 2020) and the correlation scaled differently for individual chromosomes (Takei *et al*, 2021).

## 3D ATAC‐PALM

Roughly 2‐3% of the eukaryotic genome consists of accessible regulatory elements (enhancers, promoters, insulators, etc.) crucial for cell type‐specific gene expression (Levine *et al*, 2014; Klemm *et al*, 2019). The assay for transposase‐accessible chromatin (ATAC) was developed to efficiently label accessible regions in the genome (Buenrostro *et al*, 2013). Specifically, ATAC uses a hyperactive transpose (Tn5) to insert DNA probes into accessible chromatin with high density (Adey *et al*, 2010). DNA probes can be coupled with sequencing adaptors or fluorescent dyes to map accessible chromatin sites in the linear genome (ATAC‐seq) or in the nucleus (ATAC‐see) (Buenrostro *et al*, 2013, 2015; Chen *et al*, 2016). Interestingly, ATAC‐see revealed unconventional accessible chromatin enrichment at the nuclear periphery in human neutrophil for coordinating the chromatin extrusion process underlying native immunity (Chen *et al*, 2016).

The diffraction limit, however, prohibits ATAC‐see from precisely localizing the regulatory DNA elements and reconstructing the 3D structure of the accessible genome at nanometer scales. To overcome this limitation, we recently developed a super‐resolution imaging platform, 3D ATAC‐PALM, that combined transposon biochemistry with photoactivatable Janelia Fluor 549 (PA‐JF549) (Grimm *et al*, 2016) and lattice light‐sheet microscopy (LLSM) (Xie *et al*, 2020) (Fig 2B). In this setup, the ultrathin lattice light sheet (~500 nm) (Chen *et al*, 2014a) allows efficient utilization of the photon budget for 3D single‐molecule localization by eliminating out‐of‐focus background and photo‐bleaching. 3D ATAC‐PALM overcomes the narrow axial range (1–4 µm) in other 3D SMLM techniques (Huang *et al*, 2008; Pavani *et al*, 2009; Abrahamsson *et al*, 2013). Combined with cylindrical lens‐based optical astigmatism, the high photon output of PA‐JF549 ensures precise localization (around ~20 nm in xy and ~50 nm in *z)* and 3D reconstruction of the accessible genome. Distinct from DNA FISH, gentle, and non‐denaturing ATAC labeling conditions (37°C, neutral pH) could minimize the perturbation of genome architecture.

By analyzing 3D ATAC‐PALM with multiple algorithms, we found that the accessible genome is organized into spatial clusters with characteristic size and density called accessible chromatin domains (ACDs) (Fig 2B). 3D ATAC‐PALM is compatible with multimodal imaging to simultaneously detect RNA and proteins, revealing that ACDs spatially co‐localize with active compartments, encompass actively transcribed genes, and are spatially segregated from heterochromatin. Coupled with genetic perturbation, 3D ATAC‐PALM revealed that CTCF loss leads to increased clustering and compaction of accessible chromatin, probably due to unrestrained loop extrusion by Cohesin (Xie *et al*, 2020). This result is consistent with genomic data that CTCF removal disrupts local insulation in loop domains (Nora *et al*, 2017).

## SIM

SIM surpasses the diffraction limit by illuminating the sample with structured light patterns, which generates Moiré fringes for mathematically recovering of higher frequencies (finer details) in the image (Gustafsson, 2000; Wu & Shroff, 2018). SIM typically achieves 2‐fold improvement of lateral resolution (~100 nm in xy). Further development by including a grating and a spatial light modulator improves the axial resolution and imaging speed (Gustafsson *et al*, 2008; Kner *et al*, 2009). SIM does not require labels with photo‐switching or photo‐activation properties, and multicolor imaging is much easier to implement compared with other SR techniques. These advantages have made SIM widely used in diverse biological contexts including imaging the nuclear organization.

For example, multicolor 3D‐SIM imaging of nuclear periphery resolved single nuclear pore complexes embedded within the nuclear lamina network (Schermelleh *et al*, 2008). 3D‐SIM has also been used to visualize compositional features of X chromosome (active vs inactive Barr body), network of chromatin domain clusters (a few hundred nm), and the interchromatin lacunae (Smeets *et al*, 2014). Multicolor 3D‐SIM imaging of DNA, nascent RNA, and RNA polymerase II (Pol II) revealed that a network of channels, called the interchromatin compartment, starts at nuclear pores and expands throughout the nuclear space (Markaki *et al*, 2010). Similarly, it was found that chromatin forms chain‐like reticular structures composed of chromatin domains (CDs) with a diameter of ~200–300 nm that co‐localize with putative TADs (Miron *et al*, 2020; Fig 2C). Markers of active transcription and architecture proteins tend to reside at the periphery of CDs whereas those involved in gene silencing are enriched in the interior. Likewise, nascent RNAs or associated RNPs are preferentially localized to the interchromatin lacunae outside of CDs (Miron *et al*, 2020), in agreement with results from another study (Shah *et al*, 2018). These results suggest that CDs may constitute the physical units of chromosome and potentially function as molecular “sieves” to filter nuclear machineries based on sizes to control their access to regulatory sites. More efforts should be made to characterize the relationship of CDs with putative TADs or compartmental domains (Rowley & Corces, 2018) and the physical rules underlying CD formation.

The combination of lattice light‐sheet microscopy with SIM (Chen *et al*, 2014a) could enable high speed imaging with low photo‐toxicity for probing genome organization and dynamics in living cells.

## STED

Stimulated emission depletion microscopy uses a donut shape depletion beam to induce spontaneous fluorescence emission. This effectively reduces the size of the excitation point spread function and increases the spatial resolution (Hell & Wichmann, 1994). STED retains the point‐scanning and pinhole feature of confocal microscopy while improving the resolution without post‐imaging processing like SIM or SMLM. Further technical improvement of laser pattern, speed, and commercialization has made STED a useful tool to probe complex biological systems (Vicidomini *et al*, 2018).

The tunability of STED beam to encode and retrieve spatial information in the phasor plot reduces the background and improves the resolution for imaging sub‐nuclear structures (Sarmento *et al*, 2018). STED microscopy was recently used to study genome organization protein CTCF in mouse ESCs (Gu *et al*, 2020) (Fig 2D). With a lateral resolution of ~65 nm, STED microscopy reveals that CTCF forms clusters of 2–8 molecules, with a small fraction (~25%) coupled with Cohesin. CTCF clusters are spatially co‐localized with both active (H3K4me3) and repressive (H3K27me3) histone modifications but not with RNA polymerase II, suggesting that CTCF‐mediated chromatin contact and transcription are spatially separated processes. Interestingly, transcription inhibition appeared to increase CTCF clustering, which could be reversed by Cohesin depletion, suggesting a counterbalancing role of transcription in CTCF clusters.

Recently, MINFLUX (minimizing fluorescence fluxes) microscopy was developed by combining core concepts of SMLM and STED microscopy (Balzarotti *et al*, 2017). Fluorophores are stochastically activated one at a time. Instead of using maximal photons to localize the centroid position, MINFLUX uses a doughnut‐shaped excitation beam to trace and determine where the fluorescence flux is minimal. MINFLUX has achieved localization precision down to ~1–3 nm with two orders of magnitude higher temporal resolution (~10 μs). However, MINFLUX only allows tracking of one molecule at a time, which could be harnessed to probe very fast chromatin dynamics and interactions (e.g., enhancer–promoter or transient loop anchor interactions) in live cells.

## EM

Transmission EM or scanning EM was used to image chromatin architecture *in vitro* and in fixed cells (Rouquette *et al*, 2010). However, in contrast to membrane structures (e.g., mitochondria, endoplasmic reticulum), or larger protein structures (e.g., ribosomes, condensed nucleosomes) which are highlighted by conventional stains, the phosphorus and nitrogen‐rich genomic DNA fibers are not directly labeled and appear to have poor contrast in conventional EM stains.

To overcome this challenge, serial block‐face scanning EM was combined with in situ hybridization (3D‐EMISH) and silver staining to examine the folding of a 1.7 Mbp chromatin (Trzaskoma *et al*, 2020). Another method called ChromEMT (chromatin EM tomography) was developed to employ photosensitizer (DRAQ5) to enhance DNA contrast and determine chromosome ultrastructure (Ou *et al*, 2017). DRAQ5 selectively binds to the DNA minor groove and upon photo‐activation generates highly localized, short‐lived singlet oxygen species, which react with and polymerize diaminobenzidine (DAB) for local osmium deposition, rendering DNA fibers selectively visible under EM. Because DNA within each nucleosome (~146 bp) could maximally bind ~14 DRAQ5 molecules, single nucleosomes (~10 nm) can be readily visualized. ChromEMT revealed that both interphase and mitotic chromatin are organized into disordered 5‐24 nm chains with heterogeneous local densities, challenging the traditional text‐book view of the 30 nm‐chromatin fiber as the intermediate chromatin unit *in situ* (Fig 2E).

Recently, focus‐ion beam scanning EM (FIB‐SEM) was harnessed to study the nuclear organization from Cryo‐fixed mammalian cells (Miron *et al*, 2020). The FIB‐SEM utilized iterative surface layer milling to achieve ultra‐high‐resolution 3D whole nucleus visualization at <10 nm isotropic resolution (Xu *et al*, 2017). High‐pressure freezing before freeze substitution with osmium tetroxide ensures the best possible ultrastructural preservation, revealing chromatin domains of nucleosome aggregates in the size range of ~200–300 nm (Fig 2F). However, due to the poor contrast on DNA, these high electron density regions likely reflect the histone component of heterochromatin or nucleoli observed in traditional EM studies. The average size of chromatin domain identified by this method is almost one order of magnitude larger than what was observed by ChromEMT, which could result from differences in sample preparations (cryo‐fixation vs conventional fixation) or in DNA labeling (direct imaging vs contrast enhancement). The combination of DRAQ5 staining, Cryo‐fixation, and FIB‐SEM could reveal chromatin organization in both euchromatin and heterochromatin with high fidelity.

## Correlative light and electron microscopy (CLEM)

To achieve protein‐specific imaging in the context of ultra‐structures, cryo‐3D super‐resolution (Cryo‐SR) imaging was combined with block‐face EM to examine the whole cell volume (Hoffman *et al*, 2020). Fluorescent‐labeled cells were first frozen under high pressure in vitreous ice in milliseconds to preserve cell ultrastructure, eliminating potential artifacts induced by chemical fixation (Schnell *et al*, 2012; Teves *et al*, 2016). Under cryogenic temperatures cooled by liquid helium (−265°C, 8K), typical fluorescent proteins and dyes show much reduced photo‐bleaching, increasing the photon budget stored in the sample for improving spatial resolution in 3D‐SIM and PALM imaging. Finally, the same sample was processed for 3D FIB‐SEM at 4 or 8 nm isotropic resolution followed by CLEM registration at nanometer precision.

This method was used to image chromatin structure in mouse cerebellum granule neurons and their progenitors with both euchromatin (H3.3‐SNAP‐JF552) and heterochromatin (HP1‐mEmerald) labeled (Fig 2G). Correlating the Cryo‐SIM light microscopy with FIB‐SEM data allowed the classification of chromatic regions based on known molecular markers (H3.3 or HP1). Surprisingly, the correlative analysis revealed significantly more H3.3‐enriched heterochromatin and less H3.3‐depleted euchromatin in the differentiated neurons compared with progenitor cells. This study also revealed blurred euchromatin–heterochromatin boundary during neural differentiation, arguing for the necessity of using a combination of LM and EM to dissect the mechanism of chromatin folding.

## Live cell imaging of the genome

**Figure 3:**
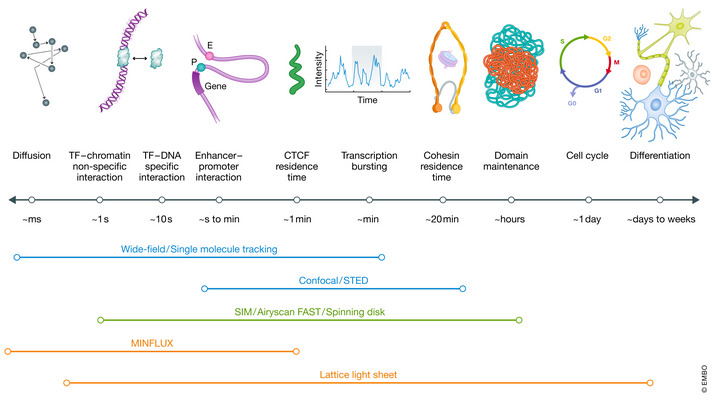
Temporal Scale of Genome Dynamics The mammalian nucleus hosts a wide range of functional events with distinct temporal kinetics. TF diffusion is fast and non‐specific chromatin collision is transient. Specific TF‐chromatin interaction usually lasts a few or tens of seconds. The enhancer to promoter interaction is very dynamic in the second’s range. The residence time for CTCF and Cohesin is ~60 s and ~20 min, respectively. Transcription bursts with frequency in the range of minutes. Chromatin domains disappear in the mitotic phase and re‐establish in the G1 phase with a lifetime of a few hours. Whereas cell cycle typically takes ~1 day, differentiation typically lasts a few days or even weeks. Wide field microscopy including those used in SMT is suited to probe the fast dynamic events reaching milliseconds resolution. Confocal microscopy and STED microscopy are based on point‐scanning and thus are slower. SIM, Airyscan FAST and spinning disk confocal have improved temporal resolution and are gentle imaging modalities for live cells. Although MINFLUX can only track one molecule at a time, it can reach a temporal resolution of microseconds, suitable for tracking very fast events. The lattice light‐sheet microscopy is particularly suited for long‐term live cell imaging.

The genome is a highly dynamic structure with functional events occurring at temporal scales spanning multiple order of magnitudes (Fig 3). Whereas the diffusion of regulatory proteins is very fast (microseconds), genomic changes associated with cell differentiation can take days or weeks. During a cell cycle, Hi‐C results show that compartments, TADs, or loops are lost in mitosis and then re‐established in G1 phase (Naumova *et al*, 2013). Even in the interphase, only ~40–50% of Cohesin and CTCF molecules engage in chromatin interactions with an average residence time ~20 min and ~1 min, respectively, suggesting that TADs and loops may dynamically form and break (Hansen *et al*, 2017). Therefore, it is imperative to use live imaging to study molecular dynamics associated with genome organization (Fig 3).

**Figure 4:**
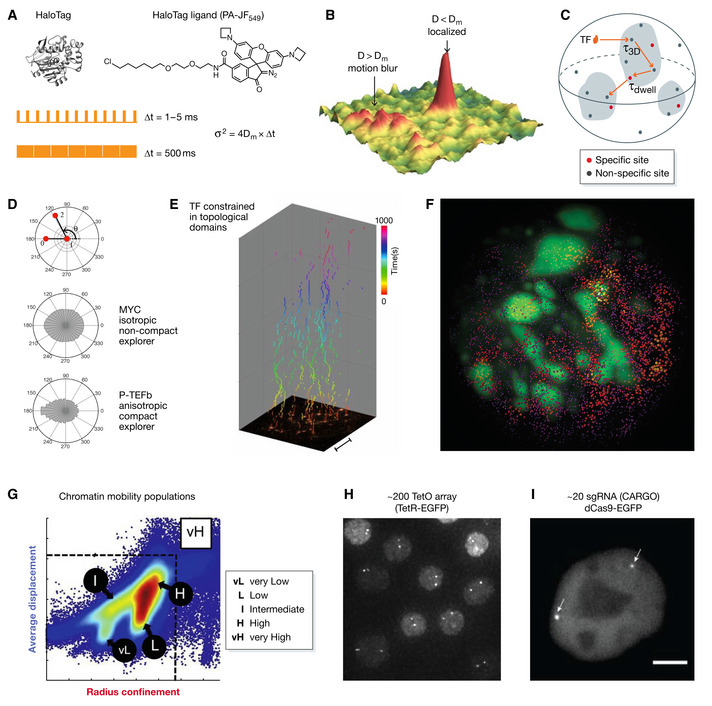
Live cell imaging of transcription factor and chromatin dynamics (A) (Upper panel) The development of self‐labeling tag (HaloTag) facilitates SMT of genome regulatory proteins inside the mammalian cell nucleus. (Lower panel). The imaging time (Δt) is tailored to the dynamics of molecules (maximal diffusion coefficient, D_m_) associated with the localization precision (σ). Given the same localization precision, higher laser power and fast camera sampling rate allowed tracking of fast‐moving molecules whereas lower laser power and slow sampling rates selectively capture less mobile molecules. (B) SMT detection sensitivity. Molecules with D smaller than the D_m_ will be localized whereas those with larger D (D > D_m_ ) will undergo motion blur and evade detection. Panel adopted from Hansen *et al* (2018) with permission. (C) Exploration of the nuclear environment by TFs probed by SMT. TFs toggle between 3D diffusion and 1D collisions. One specific binding event (t_dwell_) is interspersed with several non‐specific binding events (t_3D_). (D) Polar coordinate distribution of the angle between two consecutive translocation steps from SMT of c‐MYC and P‐TEFb. c‐MYC has isotropic distribution indicating non‐compact exploration whereas P‐TEFb shows anisotropic distribution indicating compact exploration. Adopted from Izeddin *et al* (2014) with permission. (E) Kymograph demonstrating the sparse labeling and long‐term single‐molecule tracking (up to 1,000s) of TF (e.g., Sox2). The long‐term single‐molecule imaging suggests that TF dynamically hops within restricted domains. Adopted from Liu *et al* (2018) with permission. (F) Lattice light‐sheet imaging of stable TF‐binding events, presumably enhancers, in living cells. The 3D mapped enhancers are reconstructed (red) together with heterochromatin (marked by HP1‐GFP) in a single mouse ESC. (G) SMT of histone H2B dynamics in living cells. Two‐parameter (average displacement and radius of confinement) analysis of trajectories showed distinct chromatin dynamic states. Adopted from Lerner *et al* (2020) with permission. (H) A representative image of B cells from a transgenic mouse line containing ~240 copies of tetO array knocked‐in at the IgH locus. The two IgH loci were shown as two diffraction‐limited spots. Adopted from Lucas *et al* (2014) with permission. (I) The chimeric array of gRNA oligonucleotides (CARGO) strategy was employed to assemble 12 sgRNAs into one expression cassette to label a ~2 kb segment upstream the *Fgf5* enhancer in mouse ESCs. Panel adopted from Gu *et al* (2018) with permission.

One strategy is to infer genome topology from the dynamics by which regulatory protein factors search for target sites in the nucleus. In the past decade, the development of self‐labeling tag (e.g., HaloTag) and bright, photostable, live cell compatible organic dyes (e.g., Janelia Fluor or JF dyes) (Fig 4A) has greatly facilitated live cell single‐molecule tracking (SMT) of regulatory proteins within the nucleus (Fig 4B; Liu *et al*, 2015). Transcription factors (TFs) that bind to distinct types of DNA regulatory elements have been characterized by SMT in both interphase and mitotic cells with high spatial precision (~20 nm) and fast temporal dynamics (~5–10 ms) (Mazza *et al*, 2012; Gebhardt *et al*, 2013; Chen *et al*, 2014b; Liu *et al*, 2015; Teves *et al*, 2016; Xie *et al*, 2017). A general consensus from these studies is that site‐specific TFs employ a 3D diffusion dominated search mode interspersed with many non‐specific collisions with chromatin before the relatively stable dwelling event at cognate sites (Fig 4C). The angular distribution of SMT trajectories reflects how TFs explore the local nuclear geometry. c‐MYC adopts an isotropic, non‐compact mode whereas P‐TEFb displays an anisotropic, compact search mode (Izeddin *et al*, 2014; Fig 4D). By using long‐term single‐molecule imaging based on tunable sparse labeling, we observed that TF Sox2 stably binds and dynamically hops in spatially restricted regions whereas H2B molecules are rather static, suggesting that local topological structures could sequester TF movements potentially for localized gene regulation (Liu *et al*, 2018; Fig 4E). By coupling motion blur and LLSM imaging, we systematically mapped the long‐lived Sox2‐binding events (likely cognate enhancers) in living ESCs. Interestingly, we observed that Sox2 stable binding sites form 3D clusters, spatially segregated from heterochromatin and correlated with RNA Pol II enrichment (Liu *et al*, 2014; Fig 4F). Moreover, acute depletion of the genome architectural proteins CTCF or Cohesin could significantly promote the spatial Sox2 enhancer clustering and reduce the Sox2 target search efficiency but significantly increase its dwell time (preprint: Xie *et al*, 2019), likely due to enhanced higher‐order cooperative interactions as previously discovered (Chen *et al*, 2014b; Xie *et al*, 2017).

Topologically associating domain boundaries are enriched for CTCF‐binding sites (Dixon *et al*, 2012). Deletion, inversion, or mutation of CTCF motifs disrupted chromatin loops (Sanborn *et al*, 2015) whereas ectopic insertion of CTCF sites introduced synthetic loops (Redolfi *et al*, 2019). CTCF forms spatially restricted clusters that partially correlate with Cohesin (Hansen *et al*, 2017). To understand the function of CTCF clusters, SMT reveals that CTCF exhibits significant anisotropic diffusion within a range of ~200 nm, indicating repeated site escape and bouncing back within a narrow zone. Two‐color SMT and PALM imaging suggests that such zone likely corresponds to CTCF clusters and an “anisotropy diffusion through transient trapping in zones” model was proposed to explain such dynamics. Interestingly, the RNA‐binding domain of CTCF increases the on rate of CTCF binding to chromatin and thus its target search efficiency (Hansen *et al*, 2020).

Just as the RNA‐binding domain of CTCF could accelerate its on rate kinetics, the transactivation domain of Sox2 could modulate its 3D target search and impact chromatin binding (Chen *et al*, 2014b). These observations suggest that “grammars” encoded in non‐DNA‐binding regulatory domains regulate target search kinetics. Recently, a large cohort of TFs, cofactors, and RNA polymerase II were found to contain intrinsically disordered regions (IDRs) and form locally high concentrated hubs, which are thought to compartmentalize complex and heterogeneous molecular interactions for transcription regulation (Cho *et al*, 2018; Chong *et al*, 2018; Lu *et al*, 2018; Sabari *et al*, 2018). SMT shows that proteins within the hub have significantly higher residence time (~60 s), suggesting that these hubs could increase the on rate and concomitantly decrease the off rate (Chong *et al*, 2018). This hub‐mediated stabilizing effect could potentially regulate chromatin interactions, in line with a recent theoretical predictions (Shrinivas *et al*, 2019) and mechanical chromatin fiber selection (pull‐in targets and exclude non‐targets) by a light‐inducible system (Shin *et al*, 2018).

## Live cell imaging of chromatin dynamics

Chromatin structure and dynamics could also be directly imaged in living cells. For example, live cell PALM imaging of tightly chromatin‐bound histone H2B (H2B‐PA‐mCherry) was used to examine global chromatin structure and mobility in live HeLa cells at single nucleosome resolution. It was found that chromatin exists in the form of clusters or domains with largely constant domain structure (typical radius ~110 nm) throughout different cell cycle phases and that nucleosome clusters at nuclear periphery are denser and less mobile than those at the nuclear interior (Nozaki *et al*, 2017). Although unable to distinguish distinct epigenetic states (active vs inactive), single nucleosome imaging provides global information on changes in chromatin dynamics under various perturbations. For example, inhibition of transcription elongation has an undetectable impact on chromatin structure but increases the mobility of chromatin (Nozaki *et al*, 2017; Nagashima *et al*, 2019). Considering the enrichment of transcriptional apparatus in the interchromatin lacuna (Miron *et al*, 2020), this observation suggests that the assembly of the preinitiation complex might reduce the mobility of target genes, which likely involves the hyper‐phosphorylated form of RNA Pol II. Supporting this notion, the unstructured C‐terminal repeats of the largest subunit of RNA Pol II form condensed droplet *in vitro* and quickly dissolve upon phosphorylation likely for promoter escape and transcription elongation (Boehning *et al*, 2018).

Single‐molecule tracking of histone H2B was also used to reconstruct the chromatin mobility landscape in multiple cell types (Lerner *et al*, 2020). The average displacement and radius of confinement of H2B movement were used to classify chromatin into different mobility states to discriminate their regulatory function (Fig 4G). Heterochromatin correlates with low mobility, whereas increased mobility correlates with regulatory factor binding with the notable exception of pioneering factors. An emerging theme from this study is that chromatin mobility correlates with its function. It would be interesting to perform epigenetic state‐specific chromatin labeling to further understand the nature of chromatin dynamics and function.

Locus‐specific labeling is challenging in live cells as the majority of DNA binders are in the 3D diffusion and non‐specific bound fractions, generating significant background that masks specific binding sites (Chen *et al*, 2014b; Normanno *et al*, 2015; Xie *et al*, 2017). An effective strategy is to construct repetitive DNA arrays to achieve localized signal amplification above the background. Such examples include the lac operator (LacO) array (Robinett *et al*, 1996), tet operator (TetO) array (Lucas *et al*, 2014), ANCHOR3/ParB (Germier *et al*, 2017), MS2/PP7 RNA aptamer repeats (Chen *et al*, 2018a), among others (Figure 4H). These strategies normally require non‐trivial effort of genome editing at endogenous loci. As an alternative, the CRISPR Cas9 genome‐editing system has been repurposed for genome imaging by using GFP‐tagged, catalytically dead Cas9 targeted by an array of single guide RNA (sgRNA) (Chen *et al*, 2013). The naturally existing genomic repeats and the feasibility of delivering a large amount of sgRNA make Cas9 a promising method for genome imaging in living cells. Several variants were recently developed based on engineering sgRNA structure and expression (e.g., extended loop, hybrid sgRNA; Ma *et al*, 2016, 2018; Wang *et al*, 2019) or concatemerization (Gu *et al*, 2018) (Fig 4I). Cas9 was also compatible with other signal amplification systems such as SunTag or ArrayG for long‐term imaging of specific genomic loci (Tanenbaum *et al*, 2014; Ghosh *et al*, 2019). When combined with selective plane illumination techniques such as LLSM, specific locus labeling and signal amplification could potentially enable the observation of single loci for hours and provide quantitative information on the visco‐elasticity of the nuclear environment.

## Mechanisms of genome organization

### Chromatin loops and TADs

The ring‐like Cohesin is proposed to be the primary molecular machinery driving loop formation, as Cohesin loss markedly eliminates chromatin loops (Uhlmann, 2016; Rao *et al*, 2017). As a result, factors that regulate Cohesin binding to chromatins such as CTCF, NIPBL, and WAPL also play key roles in the formation of loops and TADs (Haarhuis *et al*, 2017; Nora *et al*, 2017; Schwarzer *et al*, 2017; Wutz *et al*, 2017). 3D‐SIM imaging of individual TAD domains revealed that Cohesin facilitates intra‐TAD chromatin contacts (Szabo *et al*, 2020). On the other hand, Cohesin promotes intermingling between neighboring TADs (Luppino *et al*, 2020) while CTCF prevents this activity (Szabo *et al*, 2020). The emerging picture is that the Cohesin ring extrudes chromatin DNA and generates high probability contacts along its path until the extrusion is blocked by convergent CTCF sites at the domain boundary (Alipour & Marko, 2012; Sanborn *et al*, 2015; Fudenberg *et al*, 2016). In line with this model, super‐resolution chromatin tracing experiments uncovered that CTCF sites define the highest probability of domain boundaries (Bintu *et al*, 2018). And CTCF depletion promotes inter‐TAD contacts and increases accessible chromatin density in ACDs (Szabo *et al*, 2020; Xie *et al*, 2020).

**Figure 5:**
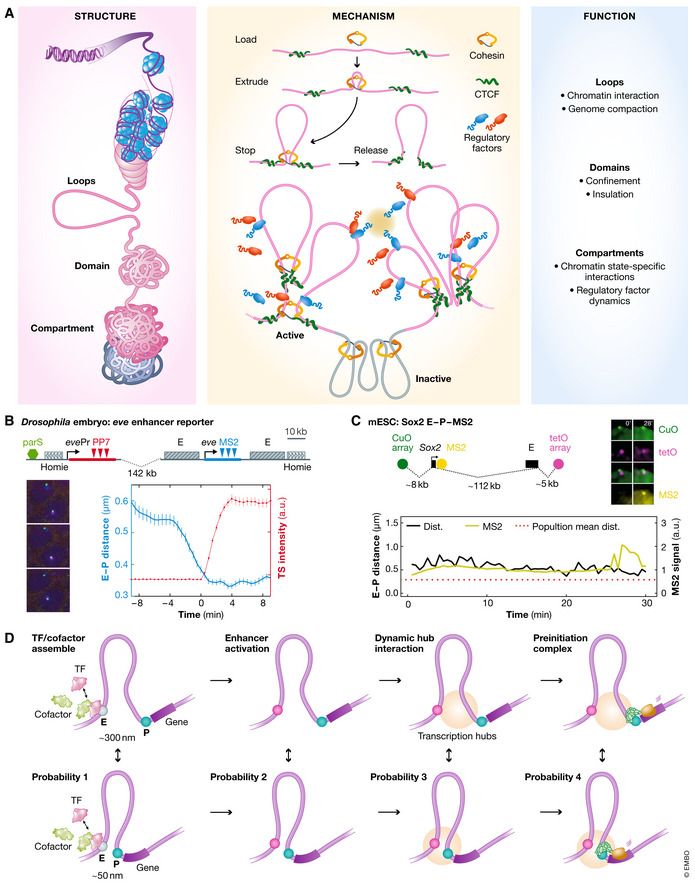
Mechanism and Function of Genome Organization (A) The putative structure–function relationships in genome organization. Loops and TADs are formed largely by Cohesin‐mediated loop extrusion. The formation of compartment is suppressed by Cohesin and likely involves abundant homo‐/hetero‐typical interactions from regulatory factors. Loops and domains could facilitate and constrain functional chromatin interactions, respectively. Compartments could regulate chromatin state‐specific interactions and regulatory factor dynamics (e.g., 3D diffusion, target search, chromatin dwelling). (B) In *Drosophila* embryo, the *eve* gene reporter has the enhancer (E, by MS2 tag), promoter (P, by ParS), and gene activity (by PP7) fluorescently labeled (upper panel). Representative E‐P interactions and gene activity are shown (lower left panel). The E‐P distance vs gene activity plot suggests the formation of E‐P loops couples with gene transcription (lower right panel). Panel adopted from Chen *et al* (2018a) with permission. (C) In mouse ESCs, the Sox2 gene enhancer (tetO), promoter (CuO), and gene activity (MS2) are simultaneously monitored (upper left panel) with representative images (upper right panel). The E‐P distance does not show obvious correlation with gene activities (lower panel). Panel adopted from Alexander *et al* (2019) with permission. (D) A putative model incorporating current evidence of chromatin topology and transcription regulation. Gene transcription is likely regulated by gene‐level chromatin topology that brings enhancers into close proximity (~300 nm) or contact (~50 nm, limited by imaging precision) with promoters. Several key regulatory steps are likely involved each with a certain probability, leading to transcriptional bursting.

Cohesin‐mediated loop extrusion process is initiated by NIPBL, followed by Cohesin translocation, and then stalled by factors such as CTCF at loop anchors (Fig 5A). NIPBL was first identified as a Cohesin loading factor. However, recent *in vitro* single‐molecule imaging experiments showed that NIPBL is essential throughout the loop extrusion process (Davidson *et al*, 2019), which is dependent on intrinsic ATPase activity of Cohesin to hydrolyze ATP as the energy source (Davidson *et al*, 2019; Kim *et al*, 2019). Interestingly, ATP is only required for the formation but not the maintenance of loops, and blocking neither transcription nor replication appears to impact loop maintenance (Vian *et al*, 2018). Consistent with these results, high‐resolution Hi‐C/Micro‐C studies recently identified “stripes” formed by Cohesin loading near high‐affinity CTCF motifs and translocating asymmetrically throughout the contact domain (Vian *et al*, 2018; Hsieh *et al*, 2020; Krietenstein *et al*, 2020).

Besides CTCF, Cohesin was also shown to interplay with other factors to regulate genome topology. For example, upon CTCF loss, Cohesin is found to position at active gene transcription start sites. Cohesin is relocated to a “Cohesin island” downstream of active genes in the absence of both CTCF and releasing factor WAPL in a transcription‐dependent manner (Ocampo‐Hafalla *et al*, 2016; Busslinger *et al*, 2017). These results suggest that RNA Pol II machinery could move Cohesin along the DNA fiber, in agreement with STED imaging result (Gu *et al*, 2020). *In vitro* single‐molecule imaging experiments suggested that nucleosomes and large‐sized DNA bound proteins are barriers to Cohesin translocation on DNA (Davidson *et al*, 2016; Stigler *et al*, 2016). It is conceivable that the physical size of certain chromatin‐associated regulatory protein complexes could modulate Cohesin extrusion and shape genome organization.

### Compartments

Compartments are maintained independently of ATP, transcription, or replication (Vian *et al*, 2018). It is also unclear whether the epigenetic state (active versus inactive) is the cause or the consequence of compartment formation. However, compartments remain after loss of loops and TADs upon Cohesin removal (Nora *et al*, 2017; Rao *et al*, 2017; Schwarzer *et al*, 2017). Similarly, super‐resolution imaging experiments found that globular “TAD‐like” domain structures, nanodomains within TADs, and chromatin domain zonations persist after Cohesin depletion (Bintu *et al*, 2018; Miron *et al*, 2020; Szabo *et al*, 2020), consistent with a Cohesin‐independent mechanism for establishing such chromatin contacts for compartmentalization.

Surprisingly, Cohesin loss even enhanced compartmental interactions resulting in more intense plaid‐like contact frequency maps and forming hundreds of links within and across chromosomes (Rao *et al*, 2017; Schwarzer *et al*, 2017). These results are consistent with a competition between loop extrusion and compartmentalization (Nuebler *et al*, 2018) but the underlying mechanisms await further studies.

The affinity interactions between heterochromatic regions have been demonstrated required for compartmentalization in heterochromatin (Falk *et al*, 2019; Mirny *et al*, 2019), and a liquid–liquid phase separation (LLPS) mechanism has been proposed. *In vitro* reconstituted nucleosome arrays could also undergo LLPS under physiological salt conditions. Linker histone H1, histone tail acetylation, and bromodomain binding all regulate the LLPS process (Gibson *et al*, 2019). Although the role of LLPS in the establishment and maintenance of chromatin compartments *in vivo* is still under debate (McSwiggen *et al*, 2019), one notion is that the concentration requirement for LLPS *in vitro* far exceeds the measured concentration *in vivo*. For instance, the measured HP1a concentration is ~3 µM in cells, far lower than the LLPS concentration requirement in vitro (40 µM). Moreover, HP1 was found to promote heterochromatin compartmentalization through specific bridging interactions without strong evidence of LLPS mechanism in live cells (Erdel *et al*, 2020). A recent study also suggests that chromatin is solid or hydrogel‐like and acts as a mesh‐like scaffold and that the liquid chromatin condensate is promoted under specific buffer conditions (i.e., reduced BSA and acetate anions; Strickfaden *et al*, 2020). It is templating to speculate that the spatial clustering of chromatin could favor hub formation with local high concentrations of homo‐ and hetero‐typical interactions involving nuclear proteins and RNA components to drive chromatin compartmentalization (Fig 5A). High‐resolution microscopy and spectroscopy experiments could provide more information on the mechanisms of genome compartmentalization *in vivo*.

## *Functional* *implications of genome structure and dynamics*

The mammalian genome stores genetic information and also bears non‐genetic functions (e.g., nuclear assembly, cell migration, nocturnal vision; Bustin & Misteli, 2016). Here, we briefly discuss the structure–function relationship between genome organization and transcription regulation.

Specifically, it is believed that chromatin loops and TADs could facilitate or constrain chromatin interactions (e.g., enhancer‐promoter, multi‐enhancer hubs), respectively (Schoenfelder & Fraser, 2019). Compartments with distinct epigenetic states and compaction level could modulate protein diffusion and target search dynamics (Fig 5A; Liu *et al*, 2014; Knight *et al*, 2015; preprint: Xie *et al*, 2019).

However, one perplexing finding is that Cohesin loss eliminates most loops and TADs but only affects the expression of a small number of genes (Rao *et al*, 2017). Conversely, transcriptional inhibition does not appear to significantly impact trans‐chromosomal or long‐range cis‐chromosomal interactions (Palstra *et al*, 2008; Su *et al*, 2020) or large chromatin architecture (e.g., TADs, compartments) (Hsieh *et al*, 2020; Jiang *et al*, 2020). A recent study identified two Cohesin populations: One is structurally associated with CTCF, while the other is dynamically associated with site‐specific TFs and transcriptional activation (Liu *et al*, 2021), suggesting distinct roles of Cohesin in genome organization and transcriptional regulation. These results suggest that large chromatin structures (e.g., TADs, compartments) do not strictly couple with transcription regulation.

Recently, nucleosome‐resolution Micro‐C detected gene‐level chromatin interactions. Transcription inhibition affected “stripes” formation originating from active promoters or enhancers (Hsieh *et al*, 2020) and increased the size of chromatin domains (Gu *et al*, 2020). It appears that, at the gene scale (kbp to tens of kbp), chromatin contacts do couple with transcription regulation, consistent with observations that RNA Pol II could translocate Cohesin ring along DNA and promote enhancer–promoter and promoter–promoter interactions (Busslinger *et al*, 2017) or CTCF clustering (Gu *et al*, 2020). These results suggest a complex interplay between genome structure and transcription regulation.

Simultaneous imaging of chromatin folding and mRNA showed that the enhancer–promoter contact is only weakly predictive of gene activation (Mateo *et al*, 2019). To rule out the possibility of temporal uncoupling, several recent studies probed the link between chromatin dynamics and transcription in living cells. In one study, transcriptional bursting kinetics (*Nanog* or *Oct4*) was analyzed by the MS2 system, while gene positioning was monitored by dCas9 targeting to the MS2 array. Interestingly, the *Nanog* locus is more mobile in the “OFF” state than in the “ON” state, whereas no such relationship is observed for *Oct4* (Ochiai *et al*, 2015). Similarly, transcription‐induced gene confinement was also observed in the cyclin D1 transgene (Germier *et al*, 2017). In addition, in a system using the MS2, ParS, and PP7 cassettes to detect the *eve* enhancer (~10 kbp apart), the promoter (~20 kbp away), and transcription activity, it was found that the proximity of enhancer to promoter (~300 nm) is required for transcriptional activation, which in‐turn promotes chromatin compaction and stabilizes the enhancer–promoter interaction (Chen *et al*, 2018a; Fig 5B). These results suggest that topological confinement is a critical step toward gene activation. Interestingly however, another study showed that the *Sox2* enhancer–promoter spatial proximity (~112‐kbp genomic separation) marked by tetO and CuO repeats is not predictive of *Sox2* gene activity in live ESCs (Alexander *et al*, 2019) (Fig 5C), in line with the report on the *Shh* locus (Benabdallah *et al*, 2019). Moreover, the *Fgf5* enhancer even displayed elevated chromatin mobility upon gene activation (Gu *et al*, 2018). Furthermore, it was found that a single enhancer can simultaneously drive synchronized bursting of two reporter genes, arguing against stable enhancer–promoter interactions by chromatin looping (Fukaya *et al*, 2016).

It is sagacious to bear in mind that the labeling (DNA (e.g., tetO, CuO, ParS) or RNA (e.g., MS2, PP7) probes) in these experiments is often ~2–8 kbp long and ~5–15 kbp away from respective enhancer or promoter. To what extent the signals represent true enhancer and promoter position awaits future studies. Ideal DNA probes should be short in length, sufficiently close to genomic features, and minimally perturbative to regulatory activities. Taken these results together, we here propose a model in which the enhancer‐based gene activation likely involves several regulatory steps each with a certain probability in a context‐dependent manner. First, site‐specific TFs dynamically engage with cis‐regulatory elements, recruiting cofactors to establish an active enhancer. Subsequently, the enhancer dynamically roams around the microenvironment and communicates with the promoter likely in the form of dynamic multivalent interactions. Finally, the preinitiation complex (e.g., general transcription factors, RNA Pol II) starts to assemble and initiates the transcription cycle (Fig 5D).

## Perspective

Using a combination of imaging and genomic strategies, significant progress has been made toward understanding the driving forces and principles underlying genome organization. However, several long‐standing challenges remain. For example, to dissect the link between chromatin dynamics, genome organization, functional output (*e.g*.*,* transcription, replication), it is necessary to implement simultaneous multicolor imaging experiments in live cells. Thus, it is critical to expand the fluorescent dye palette and develop orthogonal tags (HaloTag, SNAPTag, *etc*.) (Grimm *et al*, 2020) with high specificity and low background. Moreover, current multicolor imaging modalities mainly rely on sequential scanning, limiting the temporal resolution. It is essential to develop simultaneous multi‐channel imaging platforms by exciting dyes at different wavelengths or using dyes with large stokes shifts. As single‐molecule observation in live cells is limited by the photostability of fluorescent proteins and dyes, continued effort is required to develop new dye attachment scaffolds (e.g., FluoroCubes) with higher photostability (Niekamp *et al*, 2020).

Another challenge is to understand the relationship between genome organization and emerging structures (i.e., TF hubs) in diverse biological contexts. While CLEM is the ideal tool to address this question as demonstrated recently (Hoffman *et al*, 2020), automation is required to streamline this labor‐intensive approach. Another possible technical advance is to combine Cryo‐SR with CryoEM tomography to probe protein and genome structures in the native environment.

It is also imperative to establish high‐throughput methods to connect imaging and genomic data. Recent exciting progress in combining barcoding and automatic fluidics has overcome the scalability and throughput limitation of DNA FISH and enabled massive chromatin tracing at the scale of TADs, compartments or even whole genome in thousands of cells (Bintu *et al*, 2018; Nir *et al*, 2018; Mateo *et al*, 2019; Su *et al*, 2020; Takei *et al*, 2021). Moreover, multiplexed imaging also permits multimodal observations with transcriptional output and nuclear landmark proteins, opening up exciting opportunities to probe genome function. However, current DNA hybridization protocol still suffers from limited resolution (typically ~5–10 kbp) and harsh genome denaturation. It is crucial to develop DNA labeling techniques with high‐resolution (at sub‐kbp) to probe chromatin folding at non‐denaturing conditions to minimize genome architecture deteriorations (Solovei *et al*, 2002; Brown *et al*, 2018). 3D ATAC‐PALM could be potentially coupled with *in situ* single‐cell sequencing to gain both position and sequence information of the accessible genome in cell culture, tissue sections, and clinical specimens (Payne *et al*, 2021).

We envision that, with the techniques discussed here become standardized and routinely used, we will considerably advance our understanding of the form and function of the 3D genome in development and disease states.

## Conflict of interest

The authors declare that they have no conflict of interest.

## References

[msb20209653-bib-0001] Abrahamsson S , Chen J , Hajj B , Stallinga S , Katsov AY , Wisniewski J , Mizuguchi G , Soule P , Mueller F , Darzacq CD *et al* (2013) Fast multicolor 3D imaging using aberration‐corrected multifocus microscopy. Nat Methods 10: 60–63 23223154 10.1038/nmeth.2277 PMC4161287

[msb20209653-bib-0002] Adey A , Morrison HG , (no last name) A , Xun Xu , Kitzman JO , Turner EH , Stackhouse B , MacKenzie AP , Caruccio NC , Zhang X *et al* (2010) Rapid, low‐input, low‐bias construction of shotgun fragment libraries by high‐density in vitro transposition. Genome Biol 11: R119 21143862 10.1186/gb-2010-11-12-r119 PMC3046479

[msb20209653-bib-0003] Alexander JM , Guan J , Li B , Maliskova L , Song M , Shen Y , Huang B , Lomvardas S , Weiner OD (2019) Live‐cell imaging reveals enhancer‐dependent Sox2 transcription in the absence of enhancer proximity. Elife 8: e41769 31124784 10.7554/eLife.41769 PMC6534382

[msb20209653-bib-0004] Alipour E , Marko JF (2012) Self‐organization of domain structures by DNA‐loop‐extruding enzymes. Nucleic Acids Res 40: 11202–11212 23074191 10.1093/nar/gks925 PMC3526278

[msb20209653-bib-0005] Balzarotti F , Eilers Y , Gwosch KC , Gynnå AH , Westphal V , Stefani FD , Elf J , Hell SW (2017) Nanometer resolution imaging and tracking of fluorescent molecules with minimal photon fluxes. Science 355: 606–612 28008086 10.1126/science.aak9913

[msb20209653-bib-0006] Barbieri M , Xie SQ , Torlai Triglia E , Chiariello AM , Bianco S , De Santiago I , Branco MR , Rueda D , Nicodemi M , Pombo A (2017) Active and poised promoter states drive folding of the extended HoxB locus in mouse embryonic stem cells. Nat Struct Mol Biol 24: 515–524 28436944 10.1038/nsmb.3402

[msb20209653-bib-0007] Bates M , Blosser TR , Zhuang X (2005) Short‐Range Spectroscopic Ruler Based on a Single‐Molecule Optical Switch. Phys Rev Lett 94: 108101 15783528 10.1103/PhysRevLett.94.108101 PMC2652517

[msb20209653-bib-0008] Bates M , Huang B , Dempsey GT , Zhuang X (2007) Multicolor super‐resolution imaging with photo‐switchable fluorescent probes. Science 317: 1749–1753 17702910 10.1126/science.1146598 PMC2633025

[msb20209653-bib-0009] Beagrie RA , Scialdone A , Schueler M , Kraemer DCA , Chotalia M , Xie SQ , Barbieri M , de Santiago I , Lavitas L‐M , Branco MR *et al* (2017) Complex multi‐enhancer contacts captured by genome architecture mapping. Nature 543: 519–524 28273065 10.1038/nature21411 PMC5366070

[msb20209653-bib-0010] Beliveau BJ , Boettiger AN , Avendaño MS , Jungmann R , McCole RB , Joyce EF , Kim‐Kiselak C , Bantignies F , Fonseka CY , Erceg J *et al* (2015) Single‐molecule super‐resolution imaging of chromosomes and in situ haplotype visualization using Oligopaint FISH probes. Nat Commun 6: 7147 25962338 10.1038/ncomms8147 PMC4430122

[msb20209653-bib-0011] Beliveau BJ , Joyce EF , Apostolopoulos N , Yilmaz F , Fonseka CY , McCole RB , Chang Y , Li JB , Senaratne TN , Williams BR *et al* (2012) Versatile design and synthesis platform for visualizing genomes with Oligopaint FISH probes. Proc Natl Acad Sci 109: 21301–21306 23236188 10.1073/pnas.1213818110 PMC3535588

[msb20209653-bib-0012] Benabdallah NS , Williamson I , Illingworth RS , Kane L , Boyle S , Sengupta D , Grimes GR , Therizols P , Bickmore WA (2019) Decreased Enhancer‐Promoter Proximity Accompanying Enhancer Activation. Mol Cell 76: 473–484 31494034 10.1016/j.molcel.2019.07.038 PMC6838673

[msb20209653-bib-0013] Betzig E (1995) Proposed method for molecular optical imaging. Opt Lett 20: 237 19859146 10.1364/ol.20.000237

[msb20209653-bib-0014] Betzig E , Patterson GH , Sougrat R , Lindwasser OW , Olenych S , Bonifacino JS , Davidson MW , Lippincott‐Schwartz J , Hess HF (2006) Imaging intracellular fluorescent proteins at nanometer resolution. Science 313: 1642–1645 16902090 10.1126/science.1127344

[msb20209653-bib-0015] Bienko M , Crosetto N , Teytelman L , Klemm S , Itzkovitz S , Van Oudenaarden A (2013) A versatile genome‐scale PCR‐based pipeline for high‐definition DNA FISH. Nat Methods 10: 122–124 23263692 10.1038/nmeth.2306 PMC3735345

[msb20209653-bib-0016] Bintu B , Mateo LJ , Su JH , Sinnott‐Armstrong NA , Parker M , Kinrot S , Yamaya K , Boettiger AN , Zhuang X (2018) Super‐resolution chromatin tracing reveals domains and cooperative interactions in single cells. Science 362: eaau1783 30361340 10.1126/science.aau1783 PMC6535145

[msb20209653-bib-0017] Boehning M , Dugast‐Darzacq C , Rankovic M , Hansen AS , Yu T , Marie‐Nelly H , McSwiggen DT , Kokic G , Dailey GM , Cramer P *et al* (2018) RNA polymerase II clustering through carboxy‐terminal domain phase separation. Nat Struct Mol Biol 25: 833–840 30127355 10.1038/s41594-018-0112-y

[msb20209653-bib-0018] Boettiger AN , Bintu B , Moffitt JR , Wang S , Beliveau BJ , Fudenberg G , Imakaev M , Mirny LA , Wu CT , Zhuang X (2016) Super‐resolution imaging reveals distinct chromatin folding for different epigenetic states. Nature 529: 418–422 26760202 10.1038/nature16496 PMC4905822

[msb20209653-bib-0019] Boettiger A , Murphy S (2020) Advances in chromatin imaging at Kilobase‐scale resolution. Trends Genet 36: 273–287 32007290 10.1016/j.tig.2019.12.010 PMC7197267

[msb20209653-bib-0020] Branco MR , Pombo A (2006) Intermingling of chromosome territories in interphase suggests role in translocations and transcription‐dependent associations. PLoS Biol 4: e138 16623600 10.1371/journal.pbio.0040138 PMC1440941

[msb20209653-bib-0021] Brown JM , Roberts NA , Graham B , Waithe D , Lagerholm C , Telenius JM , De Ornellas S , Oudelaar AM , Scott C , Szczerbal I *et al* (2018) A tissue‐specific self‐interacting chromatin domain forms independently of enhancer‐promoter interactions. Nat Commun 9: 1–15 30242161 10.1038/s41467-018-06248-4 PMC6155075

[msb20209653-bib-0022] Buenrostro JD , Giresi PG , Zaba LC , Chang HY , Greenleaf WJ (2013) Transposition of native chromatin for fast and sensitive epigenomic profiling of open chromatin, DNA‐binding proteins and nucleosome position. Nat Methods 10: 1213–1218 24097267 10.1038/nmeth.2688 PMC3959825

[msb20209653-bib-0023] Buenrostro JD , Wu B , Litzenburger UM , Ruff D , Gonzales ML , Snyder MP , Chang HY , Greenleaf WJ (2015) Single‐cell chromatin accessibility reveals principles of regulatory variation. Nature 523: 486–490 26083756 10.1038/nature14590 PMC4685948

[msb20209653-bib-0024] Busslinger GA , Stocsits RR , van der Lelij P , Axelsson E , Tedeschi A , Galjart N , Peters J‐M (2017) Cohesin is positioned in mammalian genomes by transcription, CTCF and Wapl. Nature 544: 503–507 28424523 10.1038/nature22063 PMC6080695

[msb20209653-bib-0025] Bustin M , Misteli T (2016) Nongenetic functions of the genome. Science 352: aad6933 27151873 10.1126/science.aad6933 PMC6312727

[msb20209653-bib-0026] Cardozo Gizzi AM , Cattoni DI , Fiche JB , Espinola SM , Gurgo J , Messina O , Houbron C , Ogiyama Y , Papadopoulos GL , Cavalli G *et al* (2019) Microscopy‐based chromosome conformation capture enables simultaneous visualization of genome organization and transcription in intact organisms. Mol Cell 74: 212–222.e5 30795893 10.1016/j.molcel.2019.01.011

[msb20209653-bib-0027] Cardozo Gizzi AM , Espinola SM , Gurgo J , Houbron C , Fiche JB , Cattoni DI , Nollmann M (2020) Direct and simultaneous observation of transcription and chromosome architecture in single cells with Hi‐M. Nat Protoc 15: 840–876 31969721 10.1038/s41596-019-0269-9

[msb20209653-bib-0028] Chen BC , Chen B‐C , Legant WR , Legant WR , Wang K , Wang K , Shao L , Shao L , Milkie DE , Milkie DE *et al* (2014a) Lattice light‐sheet microscopy: Imaging molecules to embryos at high spatiotemporal resolution. Science 346: 1257998 25342811 10.1126/science.1257998 PMC4336192

[msb20209653-bib-0029] Chen B , Gilbert L , Cimini B , Schnitzbauer J , Zhang W , Li G‐W , Park J , Blackburn E , Weissman J , Qi L *et al* (2013) Dynamic imaging of genomic loci in living human cells by an optimized CRISPR/Cas system. Cell 155: 1479–1491 24360272 10.1016/j.cell.2013.12.001 PMC3918502

[msb20209653-bib-0030] Chen H , Levo M , Barinov L , Fujioka M , Jaynes JB , Gregor T (2018a) Dynamic interplay between enhancer–promoter topology and gene activity. Nat Genet 50: 1296–1303 30038397 10.1038/s41588-018-0175-z PMC6119122

[msb20209653-bib-0031] Chen J , Zhang Z , Li L , Chen B‐C , Revyakin A , Hajj B , Legant W , Dahan M , Lionnet T , Betzig E *et al* (2014b) Single‐molecule dynamics of enhanceosome assembly in embryonic stem cells. Cell 156: 1274–1285 24630727 10.1016/j.cell.2014.01.062 PMC4040518

[msb20209653-bib-0032] Chen X , Shen Y , Draper W , Buenrostro JD , Litzenburger U , Cho SW , Satpathy AT , Carter AC , Ghosh RP , East‐Seletsky A *et al* (2016) ATAC‐see reveals the accessible genome by transposase‐mediated imaging and sequencing. Nat Methods 13: 1013–1020 27749837 10.1038/nmeth.4031 PMC5509561

[msb20209653-bib-0033] Chen Y , Zhang Y , Wang Y , Zhang L , Brinkman EK , Adam SA , Goldman R , Van Steensel B , Ma J , Belmont AS (2018b) Mapping 3D genome organization relative to nuclear compartments using TSA‐Seq as a cytological ruler. J Cell Biol 217: 4025–4048 30154186 10.1083/jcb.201807108 PMC6219710

[msb20209653-bib-0034] Cho WK , Spille JH , Hecht M , Lee C , Li C , Grube V , Cisse II (2018) Mediator and RNA polymerase II clusters associate in transcription‐dependent condensates. Science 361: 412–415 29930094 10.1126/science.aar4199 PMC6543815

[msb20209653-bib-0035] Chong S , Dugast‐Darzacq C , Liu Z , Dong P , Dailey GM , Cattoglio C , Heckert A , Banala S , Lavis L , Darzacq X *et al* (2018) Imaging dynamic and selective low‐complexity domain interactions that control gene transcription. Science 361: eaar2555 29930090 10.1126/science.aar2555 PMC6961784

[msb20209653-bib-0036] Cremer T , Cremer C (2001) Chromosome territories, nuclear architecture and gene regulation in mammalian cells. Nat Rev Genet 2: 292–301 11283701 10.1038/35066075

[msb20209653-bib-0037] Cremer T , Cremer M (2010) Chromosome territories. Cold Spring Harb Perspect Biol 2: a003889 20300217 10.1101/cshperspect.a003889 PMC2829961

[msb20209653-bib-0038] Davidson IF , Bauer B , Goetz D , Tang W , Wutz G , Peters J‐M (2019) DNA loop extrusion by human cohesin. Science 366: 1338–1345 31753851 10.1126/science.aaz3418

[msb20209653-bib-0039] Davidson IF , Goetz D , Zaczek MP , Molodtsov MI , Huis in 't Veld PJ , Weissmann F , Litos G , Cisneros DA , Ocampo‐Hafalla M , Ladurner R *et al* (2016) Rapid movement and transcriptional re‐localization of human cohesin on DNA. EMBO J 35: 2671–2685 27799150 10.15252/embj.201695402 PMC5167347

[msb20209653-bib-0040] Dekker J , Mirny L (2016) The 3D genome as moderator of chromosomal communication. Cell 164: 1110–1121 26967279 10.1016/j.cell.2016.02.007 PMC4788811

[msb20209653-bib-0041] Dekker J , Rippe K , Dekker M , Kleckner N , Woodcock CL , Dimitrov S , Andrulis ED , Neiman AM , Zappulla DC , Sternglanz R *et al* (2002) Capturing chromosome conformation. Science 295: 1306–1311 11847345 10.1126/science.1067799

[msb20209653-bib-0042] Dixon JR , Selvaraj S , Yue F , Kim A , Li Y , Shen Y , Hu M , Liu JS , Ren B (2012) Topological domains in mammalian genomes identified by analysis of chromatin interactions. Nature 485: 376–380 22495300 10.1038/nature11082 PMC3356448

[msb20209653-bib-0043] Erdel F , Rademacher A , Vlijm R , Tünnermann J , Frank L , Weinmann R , Schweigert E , Yserentant K , Hummert J , Bauer C *et al* (2020) Mouse heterochromatin adopts digital compaction states without showing hallmarks of HP1‐driven liquid‐liquid phase separation. Mol Cell 78: 236–249 32101700 10.1016/j.molcel.2020.02.005 PMC7163299

[msb20209653-bib-0044] Falk M , Feodorova Y , Naumova N , Imakaev M , Lajoie BR , Leonhardt H , Joffe B , Dekker J , Fudenberg G , Solovei I *et al* (2019) Heterochromatin drives compartmentalization of inverted and conventional nuclei. Nature 570: 395–399 31168090 10.1038/s41586-019-1275-3 PMC7206897

[msb20209653-bib-0045] Finn EH , Pegoraro G , Brandão HB , Valton A‐L , Oomen ME , Dekker J , Mirny L , Misteli T (2019) Extensive heterogeneity and intrinsic variation in spatial genome organization. Cell 176: 1502–1515 30799036 10.1016/j.cell.2019.01.020 PMC6408223

[msb20209653-bib-0046] Fiorillo L , Musella F , Kempfer R , Chiariello AM , Bianco S , Kukalev A , Irastorza‐Azcarate I , Esposito A , Conte M , Prisco A *et al* (2020) Comparison of the Hi‐C, GAM and SPRITE methods by use of polymer models of chromatin. bioRxiv 10.1101/2020.04.24.059915 [PREPRINT]

[msb20209653-bib-0047] Fudenberg G , Imakaev M , Lu C , Goloborodko A , Abdennur N , Mirny LA (2016) Formation of chromosomal domains by loop extrusion. Cell Rep 15: 2038–2049 27210764 10.1016/j.celrep.2016.04.085 PMC4889513

[msb20209653-bib-0048] Fudenberg G , Imakaev M (2017) FISH‐ing for captured contacts: towards reconciling FISH and 3C. Nat Methods 14: 673–678 28604723 10.1038/nmeth.4329 PMC5517086

[msb20209653-bib-0049] Fukaya T , Lim B , Levine M (2016) Enhancer control of transcriptional bursting. Cell 166: 358–368 27293191 10.1016/j.cell.2016.05.025 PMC4970759

[msb20209653-bib-0050] Fussner E , Ahmed K , Dehghani H , Strauss M , Bazett‐Jones DP (2010) Changes in chromatin fiber density as a marker for pluripotency. Cold Spring Harb Symp Quant Biol 75: 245–249 21139070 10.1101/sqb.2010.75.012

[msb20209653-bib-0051] Gebhardt JCM , Suter DM , Roy R , Zhao ZW , Chapman AR , Basu S , Maniatis T , Xie XS (2013) Single‐molecule imaging of transcription factor binding to DNA in live mammalian cells. Nat Methods 10: 421–426 23524394 10.1038/nmeth.2411 PMC3664538

[msb20209653-bib-0052] Germier T , Kocanova S , Walther N , Bancaud A , Shaban HA , Sellou H , Politi AZ , Ellenberg J , Gallardo F , Bystricky K (2017) Real‐time imaging of a single gene reveals transcription‐initiated local confinement. Biophys J 113: 1383–1394 28978433 10.1016/j.bpj.2017.08.014 PMC5627313

[msb20209653-bib-0053] Ghosh RP , Franklin JM , Draper WE , Shi Q , Beltran B , Spakowitz AJ , Liphardt JT (2019) A fluorogenic array for temporally unlimited single‐molecule tracking. Nat Chem Biol 15: 401–409 30858596 10.1038/s41589-019-0241-6

[msb20209653-bib-0054] Gibson BA , Doolittle LK , Schneider MWG , Jensen LE , Gamarra N , Henry L , Gerlich DW , Redding S , Rosen MK (2019) Organization of Chromatin by Intrinsic and Regulated Phase Separation. Cell 179: 470–484.e21 31543265 10.1016/j.cell.2019.08.037 PMC6778041

[msb20209653-bib-0055] Gilbert N , Boyle S , Fiegler H , Woodfine K , Carter NP , Bickmore WA (2004) Chromatin architecture of the human genome. Cell 118: 555–566 15339661 10.1016/j.cell.2004.08.011

[msb20209653-bib-0056] Girelli G , Custodio J , Kallas T , Agostini F , Wernersson E , Spanjaard B , Mota A , Kolbeinsdottir S , Gelali E , Crosetto N *et al* (2020) GPSeq reveals the radial organization of chromatin in the cell nucleus. Nat Biotechnol 38: 1184–1193 32451505 10.1038/s41587-020-0519-y PMC7610410

[msb20209653-bib-0057] Grimm JB , English BP , Choi H , Muthusamy AK , Mehl BP , Dong P , Brown TA , Lippincott‐Schwartz J , Liu Z , Lionnet T *et al* (2016) Bright photoactivatable fluorophores for single‐molecule imaging. Nat Methods 13: 985–988. 27776112 10.1038/nmeth.4034

[msb20209653-bib-0058] Grimm JB , Tkachuk AN , Xie L , Choi H , Mohar B , Falco N , Schaefer K , Patel R , Zheng Q , Liu Z *et al* (2020) A general method to optimize and functionalize red‐shifted rhodamine dyes. Nat Methods 17: 815–821 32719532 10.1038/s41592-020-0909-6 PMC7396317

[msb20209653-bib-0059] Gu B , Comerci CJ , McCarthy DG , Saurabh S , Moerner WE , Wysocka J (2020) Opposing effects of cohesin and transcription on CTCF organization revealed by super‐resolution imaging. Mol Cell 80: 699–711 33091336 10.1016/j.molcel.2020.10.001 PMC7725164

[msb20209653-bib-0060] Gu B , Swigut T , Spencley A , Bauer MR , Chung M , Meyer T , Wysocka J (2018) Transcription‐coupled changes in nuclear mobility of mammalian cis‐regulatory elements. Science 359: 1050–1055 29371426 10.1126/science.aao3136 PMC6590518

[msb20209653-bib-0061] Gustafsson MGL (2000) Surpassing the lateral resolution limit by a factor of two using structured illumination microscopy. Short Communication. J Microsc 198: 82–87 10810003 10.1046/j.1365-2818.2000.00710.x

[msb20209653-bib-0062] Gustafsson MGL , Shao L , Carlton PM , Wang CJR , Golubovskaya IN , Cande WZ , Agard DA , Sedat JW (2008) Three‐dimensional resolution doubling in wide‐field fluorescence microscopy by structured illumination. Biophys J 94: 4957–4970 18326650 10.1529/biophysj.107.120345 PMC2397368

[msb20209653-bib-0063] Haarhuis JHI , van der Weide RH , Blomen VA , Yáñez‐Cuna JO , Amendola M , van Ruiten MS , Krijger PHL , Teunissen H , Medema RH , van Steensel B *et al* (2017) The Cohesin release factor WAPL restricts chromatin loop extension. Cell 169: 693–707 28475897 10.1016/j.cell.2017.04.013 PMC5422210

[msb20209653-bib-0064] Hansen AS , Amitai A , Cattoglio C , Tjian R , Darzacq X (2020) Guided nuclear exploration increases CTCF target search efficiency. Nat Chem Biol 16: 257–266 31792445 10.1038/s41589-019-0422-3 PMC7036004

[msb20209653-bib-0065] Hansen AS , Pustova I , Cattoglio C , Tjian R , Darzacq X (2017) CTCF and cohesin regulate chromatin loop stability with distinct dynamics. Elife 6: e25776 28467304 10.7554/eLife.25776 PMC5446243

[msb20209653-bib-0066] Hansen AS , Woringer M , Grimm JB , Lavis LD , Tjian R , Darzacq X (2018) Robust model‐based analysis of single‐particle tracking experiments with spot‐on. Elife 7: e33125 29300163 10.7554/eLife.33125 PMC5809147

[msb20209653-bib-0067] Hell SW , Wichmann J (1994) Breaking the diffraction resolution limit by stimulated emission: stimulated‐emission‐depletion fluorescence microscopy. Opt Lett 19: 780 19844443 10.1364/ol.19.000780

[msb20209653-bib-0068] Hess ST , Girirajan TPK , Mason MD (2006) Ultra‐high resolution imaging by fluorescence photoactivation localization microscopy. Biophys J 91: 4258–4272 16980368 10.1529/biophysj.106.091116 PMC1635685

[msb20209653-bib-0069] Hoffman DP , Shtengel G , Xu CS , Campbell KR , Freeman M , Wang L , Milkie DE , Pasolli HA , Iyer N , Bogovic JA *et al* (2020) Correlative three‐dimensional super‐resolution and block‐face electron microscopy of whole vitreously frozen cells. Science 367: eaaz5357 31949053 10.1126/science.aaz5357 PMC7339343

[msb20209653-bib-0070] Hsieh T‐HS , Cattoglio C , Slobodyanyuk E , Hansen AS , Rando OJ , Tjian R , Darzacq X (2020) Resolving the 3D landscape of transcription‐linked mammalian chromatin folding. Mol Cell 78: 539–553 32213323 10.1016/j.molcel.2020.03.002 PMC7703524

[msb20209653-bib-0071] Hsieh T‐HS , Weiner A , Lajoie B , Dekker J , Friedman N , Rando OJ (2015) Mapping nucleosome resolution chromosome folding in yeast by Micro‐C. Cell 162: 108–119 26119342 10.1016/j.cell.2015.05.048 PMC4509605

[msb20209653-bib-0072] Huang B , Wang W , Bates M , Zhuang X (2008) Three‐dimensional super‐resolution imaging by stochastic optical reconstruction microscopy. Science 319: 810–813 18174397 10.1126/science.1153529 PMC2633023

[msb20209653-bib-0073] Huff J (2015) The Airyscan detector from ZEISS: confocal imaging with improved signal‐to‐noise ratio and super‐resolution. Nat Methods 12: i–ii

[msb20209653-bib-0074] Izeddin I , Récamier V , Bosanac L , Cissé II , Boudarene L , Dugast‐Darzacq C , Proux F , Bénichou O , Voituriez R , Bensaude O *et al* (2014) Single‐molecule tracking in live cells reveals distinct target‐search strategies of transcription factors in the nucleus. Elife 3: e02230 10.7554/eLife.02230 PMC4095940 24925319

[msb20209653-bib-0075] Jiang Y , Huang J , Lun K , Li B , Zheng H , Li Y , Zhou R , Duan W , Wang C , Feng Y *et al* (2020) Genome‐wide analyses of chromatin interactions after the loss of Pol I, Pol II, and Pol III. Genome Biol 21: 158 32616013 10.1186/s13059-020-02067-3 PMC7331254

[msb20209653-bib-0076] Jungmann R , Steinhauer C , Scheible M , Kuzyk A , Tinnefeld P , Simmel FC (2010) Single‐molecule kinetics and super‐resolution microscopy by fluorescence imaging of transient binding on DNA origami. Nano Lett 10: 4756–4761 20957983 10.1021/nl103427w

[msb20209653-bib-0077] Kempfer R , Pombo A (2020) Methods for mapping 3D chromosome architecture. Nat Rev Genet 21: 207–226 31848476 10.1038/s41576-019-0195-2

[msb20209653-bib-0078] Kim Y , Shi Z , Zhang H , Finkelstein IJ , Yu H (2019) Human cohesin compacts DNA by loop extrusion. Science 366: 1345–1349 31780627 10.1126/science.aaz4475 PMC7387118

[msb20209653-bib-0079] Klemm SL , Shipony Z , Greenleaf WJ (2019) Chromatin accessibility and the regulatory epigenome. Nat Rev Genet 20: 207–220 30675018 10.1038/s41576-018-0089-8

[msb20209653-bib-0080] Kner P , Chhun BB , Griffis ER , Winoto L , Gustafsson MGL (2009) Super‐resolution video microscopy of live cells by structured illumination. Nat Methods 6: 339–342 19404253 10.1038/nmeth.1324 PMC2895555

[msb20209653-bib-0081] Knight SC , Xie L , Deng W , Guglielmi B , Witkowsky LB , Bosanac L , Zhang ET , El Beheiry M , Masson J‐B , Dahan M *et al* (2015) Dynamics of CRISPR‐Cas9 genome interrogation in living cells. Science 350: 823–826 26564855 10.1126/science.aac6572

[msb20209653-bib-0082] Krietenstein N , Abraham S , Venev SV , Abdennur N , Gibcus J , Hsieh T‐H , Parsi KM , Yang L , Maehr R , Mirny LA *et al* (2020) Ultrastructural details of mammalian chromosome architecture. Mol Cell 78: 554–565 32213324 10.1016/j.molcel.2020.03.003 PMC7222625

[msb20209653-bib-0083] Legant WR , Shao L , Grimm JB , Brown TA , Milkie DE , Avants BB , Lavis LD , Betzig E (2016) High‐density three‐dimensional localization microscopy across large volumes. Nat Methods 13: 359–365 26950745 10.1038/nmeth.3797 PMC4889433

[msb20209653-bib-0084] Lerner J , Gomez‐Garcia PA , McCarthy RL , Liu Z , Lakadamyali M , Zaret KS (2020) Two‐parameter mobility assessments discriminate diverse regulatory factor behaviors in chromatin. Mol Cell 79: 677–688 32574554 10.1016/j.molcel.2020.05.036 PMC7483934

[msb20209653-bib-0085] Levine M , Cattoglio C , Tjian R (2014) Looping back to leap forward: Transcription enters a new era. Cell 157: 13–25 24679523 10.1016/j.cell.2014.02.009 PMC4059561

[msb20209653-bib-0086] Lieberman‐Aiden E , van Berkum Nl , Williams L , Imakaev M , Ragoczy T , Telling A , Amit I , Lajoie Br , Sabo Pj , Dorschner Mo *et al* (2009) Comprehensive mapping of long‐range interactions reveals folding principles of the human genome. Science 326: 289–293 19815776 10.1126/science.1181369 PMC2858594

[msb20209653-bib-0087] Liu H , Dong P , Ioannou MS , Li Li , Shea J , Pasolli HA , Grimm JB , Rivlin PK , Lavis LD , Koyama M *et al* (2018) Visualizing long‐term single‐molecule dynamics in vivo by stochastic protein labeling. Proc Natl Acad Sci 115: 343–348 29284749 10.1073/pnas.1713895115 PMC5777047

[msb20209653-bib-0088] Liu NQ , Maresca M , van den Brand T , Braccioli L , Schijns MMGA , Teunissen H , Bruneau BG , Nora EP , de Wit E (2021) WAPL maintains a cohesin loading cycle to preserve cell‐type‐specific distal gene regulation. Nat Genet 53: 100–109 33318687 10.1038/s41588-020-00744-4 PMC7610352

[msb20209653-bib-0089] Liu Z , Lavis LD , Betzig E (2015) Imaging live‐cell dynamics and structure at the single‐molecule level. Mol Cell 58: 644–659 26000849 10.1016/j.molcel.2015.02.033

[msb20209653-bib-0090] Liu Z , Legant WR , Chen B‐C , Li L , Grimm JB , Lavis LD , Betzig E , Tjian R (2014) 3D imaging of Sox2 enhancer clusters in embryonic stem cells. Elife 3: 1–29 10.7554/eLife.04236 PMC4381973 25537195

[msb20209653-bib-0091] Loviglio Mn , Leleu M , Männik K , Passeggeri M , Giannuzzi G , van der Werf I , Waszak Sm , Zazhytska M , Roberts‐Caldeira I , Gheldof N *et al* (2017) Chromosomal contacts connect loci associated with autism, BMI and head circumference phenotypes. Mol Psychiatry 22: 836–849 27240531 10.1038/mp.2016.84 PMC5508252

[msb20209653-bib-0092] Lu H , Yu D , Hansen AS , Ganguly S , Liu R , Heckert A , Darzacq X , Zhou Q (2018) Phase‐separation mechanism for C‐terminal hyperphosphorylation of RNA polymerase II. Nature 558: 318–323 29849146 10.1038/s41586-018-0174-3 PMC6475116

[msb20209653-bib-0093] Lucas JS , Zhang Y , Dudko OK , Murre C (2014) 3D trajectories adopted by coding and regulatory DNA elements: first‐passage times for genomic interactions. Cell 158: 339–352 24998931 10.1016/j.cell.2014.05.036 PMC4113018

[msb20209653-bib-0094] Luppino JM , Park DS , Nguyen SC , Lan Y , Xu Z , Yunker R , Joyce EF (2020) Cohesin promotes stochastic domain intermingling to ensure proper regulation of boundary‐proximal genes. Nat Genet 52: 840–848 32572210 10.1038/s41588-020-0647-9 PMC7416539

[msb20209653-bib-0095] Ma H , Tu L‐C , Naseri A , Huisman M , Zhang S , Grunwald D , Pederson T (2016) Multiplexed labeling of genomic loci with dCas9 and engineered sgRNAs using CRISPRainbow. Nat Biotechnol 34: 528–530 27088723 10.1038/nbt.3526 PMC4864854

[msb20209653-bib-0096] Ma H , Tu L‐C , Naseri A , Chung Y‐C , Grunwald D , Zhang S , Pederson T (2018) CRISPR‐Sirius: RNA scaffolds for signal amplification in genome imaging. Nat Methods 15: 928–931 30377374 10.1038/s41592-018-0174-0 PMC6252086

[msb20209653-bib-0097] Markaki Y , Gunkel M , Schermelleh L , Beichmanis S , Neumann J , Heidemann M , Leonhardt H , Eick D , Cremer C , Cremer T (2010) Functional nuclear organization of transcription and DNA replication: a topographical marriage between chromatin domains and the interchromatin compartment. Cold Spring Harb Symp Quant Biol 75: 475–492 21467142 10.1101/sqb.2010.75.042

[msb20209653-bib-0098] Mateo LJ , Murphy SE , Hafner A , Cinquini IS , Walker CA , Boettiger AN (2019) Visualizing DNA folding and RNA in embryos at single‐cell resolution. Nature 568: 49–54 30886393 10.1038/s41586-019-1035-4 PMC6556380

[msb20209653-bib-0099] Mazza D , Abernathy A , Golob N , Morisaki T , McNally JG (2012) A benchmark for chromatin binding measurements in live cells. Nucleic Acids Res 40: e119–e119 22844090 10.1093/nar/gks701 PMC3424588

[msb20209653-bib-0100] McCord RP , Kaplan N , Giorgetti L (2020) Chromosome conformation capture and beyond: toward an integrative view of chromosome structure and function. Mol Cell 77: 688–708 32001106 10.1016/j.molcel.2019.12.021 PMC7134573

[msb20209653-bib-0101] McSwiggen DT , Mir M , Darzacq X , Tjian R (2019) Evaluating phase separation in live cells: diagnosis, caveats, and functional consequences. Genes Dev 33: 1619–1634 31594803 10.1101/gad.331520.119 PMC6942051

[msb20209653-bib-0102] Mirny LA , Imakaev M , Abdennur N (2019) Two major mechanisms of chromosome organization. Curr Opin Cell Biol 58: 142–152 31228682 10.1016/j.ceb.2019.05.001 PMC6800258

[msb20209653-bib-0103] Miron E , Oldenkamp R , Brown JM , Pinto DMS , Xu CS , Faria AR , Shaban HA , Rhodes JDP , Innocent C , de Ornellas S *et al* (2020) Chromatin arranges in chains of mesoscale domains with nanoscale functional topography independent of cohesin. Sci Adv 6: eaba8811 32967822 10.1126/sciadv.aba8811 PMC7531892

[msb20209653-bib-0104] Monahan K , Horta A , Lomvardas S (2019) LHX2‐ and LDB1‐mediated trans interactions regulate olfactory receptor choice. Nature 565: 448–453 30626972 10.1038/s41586-018-0845-0 PMC6436840

[msb20209653-bib-0105] Mumbach MR , Rubin AJ , Flynn RA , Dai C , Khavari PA , Greenleaf WJ , Chang HY (2016) HiChIP: efficient and sensitive analysis of protein‐directed genome architecture. Nat Methods 13: 919–922 27643841 10.1038/nmeth.3999 PMC5501173

[msb20209653-bib-0106] Nagashima R , Hibino K , Ashwin Ss , Babokhov M , Fujishiro S , Imai R , Nozaki T , Tamura S , Tani T , Kimura H *et al* (2019) Single nucleosome imaging reveals loose genome chromatin networks via active RNA polymerase II. J Cell Biol 218: 1511–1530 30824489 10.1083/jcb.201811090 PMC6504897

[msb20209653-bib-0107] Naumova N , Imakaev M , Fudenberg G , Zhan Y , Lajoie BR , Mirny LA , Dekker J (2013) Organization of the Mitotic Chromosome. Science 342: 948–953 24200812 10.1126/science.1236083 PMC4040465

[msb20209653-bib-0108] Niekamp S , Stuurman N , Vale RD (2020) A 6‐nm ultra‐photostable DNA FluoroCube for fluorescence imaging. Nat Methods 17: 437–441 32203385 10.1038/s41592-020-0782-3 PMC7138518

[msb20209653-bib-0109] Nir G , Farabella I , Pérez Estrada C , Ebeling CG , Beliveau BJ , Sasaki HM , Lee SD , Nguyen SC , McCole RB , Chattoraj S *et al* (2018) Walking along chromosomes with super‐resolution imaging, contact maps, and integrative modeling. PLOS Genet 14: e1007872 30586358 10.1371/journal.pgen.1007872 PMC6324821

[msb20209653-bib-0110] Nora EP , Goloborodko A , Valton AL , Gibcus JH , Uebersohn A , Abdennur N , Dekker J , Mirny LA , Bruneau BG (2017) Targeted degradation of CTCF decouples local insulation of chromosome domains from genomic compartmentalization. Cell 169: 930–944 28525758 10.1016/j.cell.2017.05.004 PMC5538188

[msb20209653-bib-0111] Nora EP , Lajoie BR , Schulz EG , Giorgetti L , Okamoto I , Servant N , Piolot T , van Berkum NL , Meisig J , Sedat J *et al* (2012) Spatial partitioning of the regulatory landscape of the X‐inactivation centre. Nature 485: 381–385 22495304 10.1038/nature11049 PMC3555144

[msb20209653-bib-0112] Normanno D , Boudarène L , Dugast‐Darzacq C , Chen J , Richter C , Proux F , Bénichou O , Voituriez R , Darzacq X , Dahan M (2015) Probing the target search of DNA‐binding proteins in mammalian cells using TetR as model searcher. Nat Commun 6: 7357 26151127 10.1038/ncomms8357 PMC4507003

[msb20209653-bib-0113] Nozaki T , Imai R , Tanbo M , Nagashima R , Tamura S , Tani T , Joti Y , Tomita M , Hibino K , Kanemaki MT *et al* (2017) Dynamic organization of chromatin domains revealed by super‐resolution live‐cell imaging. Mol Cell 67: 282–293.e7 28712725 10.1016/j.molcel.2017.06.018

[msb20209653-bib-0114] Nuebler J , Fudenberg G , Imakaev M , Abdennur N , Mirny LA (2018) Chromatin organization by an interplay of loop extrusion and compartmental segregation. Proc Natl Acad Sci 115: E6697–E6706 29967174 10.1073/pnas.1717730115 PMC6055145

[msb20209653-bib-0115] Ocampo‐Hafalla M , Muñoz S , Samora CP , Uhlmann F (2016) Evidence for cohesin sliding along budding yeast chromosomes. Open Biol 6: 150178 27278645 10.1098/rsob.150178 PMC4929932

[msb20209653-bib-0116] Ochiai H , Sugawara T , Yamamoto T (2015) Simultaneous live imaging of the transcription and nuclear position of specific genes. Nucleic Acids Res 43: e127 26092696 10.1093/nar/gkv624 PMC4627063

[msb20209653-bib-0117] Ou HD , Phan S , Deerinck TJ , Thor A , Ellisman MH , O’Shea CC (2017) ChromEMT: Visualizing 3D chromatin structure and compaction in interphase and mitotic cells. Science 357: eaag0025 28751582 10.1126/science.aag0025 PMC5646685

[msb20209653-bib-0118] Palstra R‐J , Simonis M , Klous P , Brasset E , Eijkelkamp B , de Laat W (2008) Maintenance of long‐range DNA interactions after inhibition of ongoing RNA polymerase II transcription. PLoS One 3: e1661 18286208 10.1371/journal.pone.0001661 PMC2243019

[msb20209653-bib-0119] Patterson GH (2002) A Photoactivatable GFP for Selective Photolabeling of Proteins and Cells. Science 297: 1873–1877 12228718 10.1126/science.1074952

[msb20209653-bib-0120] Pavani SRP , Thompson MA , Biteen JS , Lord SJ , Liu N , Twieg RJ , Piestun R , Moerner WE (2009) Three‐dimensional, single‐molecule fluorescence imaging beyond the diffraction limit by using a double‐helix point spread function. Proc Natl Acad Sci 106: 2995–2999 19211795 10.1073/pnas.0900245106 PMC2651341

[msb20209653-bib-0121] Payne AC , Chiang ZD , Reginato PL , Mangiameli SM , Murray EM , Yao C‐C , Markoulaki S , Earl AS , Labade AS , Jaenisch R *et al* (2021) In situ genome sequencing resolves DNA sequence and structure in intact biological samples. Science 371: eaay3446 33384301 10.1126/science.aay3446 PMC7962746

[msb20209653-bib-0122] Quinodoz SA , Ollikainen N , Tabak B , Palla A , Schmidt JM , Detmar E , Lai MM , Shishkin AA , Bhat P , Takei Y *et al* (2018) Higher‐order inter‐chromosomal hubs shape 3D genome organization in the nucleus. Cell 174: 744–757 29887377 10.1016/j.cell.2018.05.024 PMC6548320

[msb20209653-bib-0123] Rao SSP , Huang S‐C , Glenn St Hilaire B , Engreitz JM , Perez EM , Kieffer‐Kwon K‐R , Sanborn AL , Johnstone SE , Bascom GD , Bochkov ID *et al* (2017) Cohesin loss eliminates all loop domains. Cell 171: 305–320 28985562 10.1016/j.cell.2017.09.026 PMC5846482

[msb20209653-bib-0124] Rao S , Huntley M , Durand N , Stamenova E , Bochkov I , Robinson J , Sanborn A , Machol I , Omer A , Lander E *et al* (2014) A 3D map of the human genome at kilobase resolution reveals principles of chromatin looping. Cell 159: 1665–1680 25497547 10.1016/j.cell.2014.11.021 PMC5635824

[msb20209653-bib-0125] Redolfi J , Zhan Y , Valdes‐Quezada C , Kryzhanovska M , Guerreiro I , Iesmantavicius V , Pollex T , Grand RS , Mulugeta E , Kind J *et al* (2019) DamC reveals principles of chromatin folding in vivo without crosslinking and ligation. Nat Struct Mol Biol 26: 471–480 31133702 10.1038/s41594-019-0231-0 PMC6561777

[msb20209653-bib-0126] Ricci MA , Manzo C , García‐Parajo MF , Lakadamyali M , Cosma MP (2015) Chromatin fibers are formed by heterogeneous groups of nucleosomes in vivo. Cell 160: 1145–1158 25768910 10.1016/j.cell.2015.01.054

[msb20209653-bib-0127] Robinett CC , Straight A , Li G , Willhelm C , Sudlow G , Murray A , Belmont AS (1996) In vivo localization of DNA sequences and visualization of large‐scale chromatin organization using lac operator/repressor recognition. J Cell Biol 135: 1685–1700 8991083 10.1083/jcb.135.6.1685 PMC2133976

[msb20209653-bib-0128] Rouquette J , Cremer C , Cremer T , Fakan S (2010) Functional nuclear architecture studied by microscopy: present and future. Int Rev Cell Mol Biol 282: 1–90 20630466 10.1016/S1937-6448(10)82001-5

[msb20209653-bib-0129] Rowley MJ , Corces VG (2018) Organizational principles of 3D genome architecture. Nat Rev Genet 19: 789–800 30367165 10.1038/s41576-018-0060-8 PMC6312108

[msb20209653-bib-0130] Rust MJ , Bates M , Zhuang X (2006) Sub‐diffraction‐limit imaging by stochastic optical reconstruction microscopy (STORM). Nat Methods 3: 793–796 16896339 10.1038/nmeth929 PMC2700296

[msb20209653-bib-0131] Sabari BR , Dall’Agnese A , Boija A , Klein IA , Coffey EL , Shrinivas K , Abraham BJ , Hannett NM , Zamudio AV , Manteiga JC *et al* (2018) Coactivator condensation at super‐enhancers links phase separation and gene control. Science 361: 387–392 29930091 10.1126/science.aar3958 PMC6092193

[msb20209653-bib-0132] Sanborn AL , Rao SSP , Huang S‐C , Durand NC , Huntley MH , Jewett AI , Bochkov ID , Chinnappan D , Cutkosky A , Li J *et al* (2015) Chromatin extrusion explains key features of loop and domain formation in wild‐type and engineered genomes. Proc Natl Acad Sci 112: E6456–E6465 26499245 10.1073/pnas.1518552112 PMC4664323

[msb20209653-bib-0133] Sarmento MJ , Oneto M , Pelicci S , Pesce L , Scipioni L , Faretta M , Furia L , Dellino GI , Pelicci PG , Bianchini P *et al* (2018) Exploiting the tunability of stimulated emission depletion microscopy for super‐resolution imaging of nuclear structures. Nat Commun 9: 3415 30143630 10.1038/s41467-018-05963-2 PMC6109149

[msb20209653-bib-0134] Schermelleh L , Carlton Pm , Haase S , Shao L , Winoto L , Kner P , Burke B , Cardoso Mc , Agard Da , Gustafsson M *et al* (2008) Subdiffraction multicolor imaging of the nuclear periphery with 3D structured illumination microscopy. Science 320: 1332–1336 18535242 10.1126/science.1156947 PMC2916659

[msb20209653-bib-0135] Schnell U , Dijk F , Sjollema KA , Giepmans BNG (2012) Immunolabeling artifacts and the need for live‐cell imaging. Nat Methods. 9: 152–158 22290187 10.1038/nmeth.1855

[msb20209653-bib-0136] Schoenfelder S , Fraser P (2019) Long‐range enhancer–promoter contacts in gene expression control. Nat Rev Genet 20: 437–455 31086298 10.1038/s41576-019-0128-0

[msb20209653-bib-0137] Schwarzer W , Abdennur N , Goloborodko A , Pekowska A , Fudenberg G , Loe‐Mie Y , Fonseca NA , Huber W , Haering CH , Mirny L *et al* (2017) Two independent modes of chromatin organization revealed by cohesin removal. Nature 551: 51–56 29094699 10.1038/nature24281 PMC5687303

[msb20209653-bib-0138] Sexton T , Yaffe E , Kenigsberg E , Bantignies F , Leblanc B , Hoichman M , Parrinello H , Tanay A , Cavalli G (2012) Three‐dimensional folding and functional organization principles of the Drosophila genome. Cell 148: 458–472 22265598 10.1016/j.cell.2012.01.010

[msb20209653-bib-0139] Shachar S , Voss TC , Pegoraro G , Sciascia N , Misteli T (2015) Identification of gene positioning factors using high‐throughput imaging mapping. Cell 162: 911–923 26276637 10.1016/j.cell.2015.07.035 PMC4538709

[msb20209653-bib-0140] Shah S , Takei Y , Zhou W , Lubeck E , Yun J , Eng C‐H , Koulena N , Cronin C , Karp C , Liaw EJ *et al* (2018) Dynamics and spatial genomics of the nascent transcriptome by intron seqFISH. Cell 174: 363–376 29887381 10.1016/j.cell.2018.05.035 PMC6046268

[msb20209653-bib-0141] Shin Y , Chang YC , Lee DSW , Berry J , Sanders DW , Ronceray P , Wingreen NS , Haataja M , Brangwynne CP (2018) Liquid nuclear condensates mechanically sense and restructure the genome. Cell 175: 1481–1491 30500535 10.1016/j.cell.2018.10.057 PMC6724728

[msb20209653-bib-0142] Shrinivas K , Sabari BR , Coffey EL , Klein IA , Boija A , Zamudio AV , Schuijers J , Hannett NM , Sharp PA , Young RA *et al* (2019) Enhancer features that drive formation of transcriptional condensates. Mol Cell 75: 549–561 31398323 10.1016/j.molcel.2019.07.009 PMC6690378

[msb20209653-bib-0143] Simonis M , Klous P , Splinter E , Moshkin Y , Willemsen R , De Wit E , Van Steensel B , De Laat W (2006) Nuclear organization of active and inactive chromatin domains uncovered by chromosome conformation capture‐on‐chip (4C). Nat Genet 38: 1348–1354 17033623 10.1038/ng1896

[msb20209653-bib-0144] Smeets D , Markaki Y , Schmid VJ , Kraus F , Tattermusch A , Cerase A , Sterr M , Fiedler S , Demmerle J , Popken J *et al* (2014) Three‐dimensional super‐resolution microscopy of the inactive X chromosome territory reveals a collapse of its active nuclear compartment harboring distinct Xist RNA foci. Epigenetics Chromatin 7: 8 25057298 10.1186/1756-8935-7-8 PMC4108088

[msb20209653-bib-0145] Solovei I , Cavallo A , Schermelleh L , Jaunin F , Scasselati C , Cmarko D , Cremer C , Fakan S , Cremer T (2002) Spatial preservation of nuclear chromatin architecture during three‐dimensional fluorescence in situ hybridization (3D‐FISH). Exp Cell Res 276: 10–23 11978004 10.1006/excr.2002.5513

[msb20209653-bib-0146] Solovei I , Cremer M (2010) 3D‐FISH on cultured cells combined with immunostaining. Methods Mol Biol 659: 117–126 20809307 10.1007/978-1-60761-789-1_8

[msb20209653-bib-0147] van Steensel B , Belmont AS (2017) Lamina‐associated domains: links with chromosome architecture, heterochromatin, and gene repression. Cell 169: 780–791 28525751 10.1016/j.cell.2017.04.022 PMC5532494

[msb20209653-bib-0148] Stevens TJ , Lando D , Basu S , Atkinson LP , Cao Y , Lee SF , Leeb M , Wohlfahrt KJ , Boucher W , O’Shaughnessy‐Kirwan A *et al* (2017) 3D structures of individual mammalian genomes studied by single‐cell Hi‐C. Nature 544: 59–64 28289288 10.1038/nature21429 PMC5385134

[msb20209653-bib-0149] Stigler J , Çamdere GÖ , Koshland DE , Greene EC (2016) Single‐molecule imaging reveals a collapsed conformational state for DNA‐bound cohesin. Cell Rep 15: 988–998 27117417 10.1016/j.celrep.2016.04.003 PMC4856582

[msb20209653-bib-0150] Strickfaden H , Tolsma TO , Sharma A , Underhill DA , Hansen JC , Hendzel MJ (2020) Condensed chromatin behaves like a solid on the mesoscale in vitro and in living cells. Cell 183: 1772–1784 33326747 10.1016/j.cell.2020.11.027

[msb20209653-bib-0151] Su J‐H , Zheng P , Kinrot SS , Bintu B , Zhuang X (2020) Genome‐scale imaging of the 3D organization and transcriptional activity of chromatin. Cell 182: 1641–1659 32822575 10.1016/j.cell.2020.07.032 PMC7851072

[msb20209653-bib-0152] Szabo Q , Donjon A , Jerković I , Papadopoulos GL , Cheutin T , Bonev B , Nora EP , Bruneau BG , Bantignies F , Cavalli G (2020) Regulation of single‐cell genome organization into TADs and chromatin nanodomains. Nat Genet 52: 1151–1157 33077913 10.1038/s41588-020-00716-8 PMC7610512

[msb20209653-bib-0153] Szabo Q , Jost D , Chang J‐M , Cattoni DI , Papadopoulos GL , Bonev B , Sexton T , Gurgo J , Jacquier C , Nollmann M *et al* (2018) TADs are 3D structural units of higher‐order chromosome organization in Drosophila. Sci Adv 4: eaar8082 29503869 10.1126/sciadv.aar8082 PMC5829972

[msb20209653-bib-0154] Takei Y , Yun J , Zheng S , Ollikainen N , Pierson N , White J , Shah S , Thomassie J , Suo S , Eng C‐H *et al* (2021) Integrated spatial genomics reveals global architecture of single nuclei. Nature 590: 344–350 33505024 10.1038/s41586-020-03126-2 PMC7878433

[msb20209653-bib-0155] Tan L , Xing D , Chang C‐H , Li H , Xie XS (2018) Three‐dimensional genome structures of single diploid human cells. Science 361: 924–928 30166492 10.1126/science.aat5641 PMC6360088

[msb20209653-bib-0156] Tanenbaum ME , Gilbert LA , Qi LS , Weissman JS , Vale RD (2014) A protein‐tagging system for signal amplification in gene expression and fluorescence imaging. Cell 159: 635–646 25307933 10.1016/j.cell.2014.09.039 PMC4252608

[msb20209653-bib-0157] Tang Z , Luo O , Li X , Zheng M , Zhu J , Szalaj P , Trzaskoma P , Magalska A , Wlodarczyk J , Ruszczycki B *et al* (2015) CTCF‐mediated human 3D genome architecture reveals chromatin topology for transcription. Cell 163: 1611–1627 26686651 10.1016/j.cell.2015.11.024 PMC4734140

[msb20209653-bib-0158] Teves SS , An L , Hansen AS , Xie L , Darzacq X , Tjian R (2016) A dynamic mode of mitotic bookmarking by transcription factors. Elife 5: e22280 10.7554/eLife.22280 PMC5156526 27855781

[msb20209653-bib-0159] Trzaskoma P , Ruszczycki B , Lee B , Pels KK , Krawczyk K , Bokota G , Szczepankiewicz AA , Aaron J , Walczak A , Śliwińska MA *et al* (2020) Ultrastructural visualization of 3D chromatin folding using volume electron microscopy and DNA in situ hybridization. Nat Commun 11: 1–9 32358536 10.1038/s41467-020-15987-2 PMC7195386

[msb20209653-bib-0160] Uhlmann F (2016) SMC complexes: From DNA to chromosomes. Nat Rev Mol Cell Biol 17: 399–412 27075410 10.1038/nrm.2016.30

[msb20209653-bib-0161] Vian L , Pękowska A , Rao SSP , Kieffer‐Kwon K‐R , Jung S , Baranello L , Huang S‐C , El Khattabi L , Dose M , Pruett N *et al* (2018) The energetics and physiological impact of cohesin extrusion. Cell 173: 1165–1178 29706548 10.1016/j.cell.2018.03.072 PMC6065110

[msb20209653-bib-0162] Vicidomini G , Bianchini P , Diaspro A (2018) STED super‐resolved microscopy. Nat Methods 15: 173–182 29377014 10.1038/nmeth.4593

[msb20209653-bib-0163] Wang H , Nakamura M , Abbott TR , Zhao D , Luo K , Yu C , Nguyen CM , Lo A , Daley TP , La Russa M *et al* (2019) CRISPR‐mediated live imaging of genome editing and transcription. Science 365: 1301–1305 31488703 10.1126/science.aax7852 PMC13030913

[msb20209653-bib-0164] Wang S , Su JH , Beliveau BJ , Bintu B , Moffitt JR , Wu CT , Zhuang X (2016) Spatial organization of chromatin domains and compartments in single chromosomes. Science 353: 598–602 27445307 10.1126/science.aaf8084 PMC4991974

[msb20209653-bib-0165] Williamson I , Berlivet S , Eskeland R , Boyle S , Illingworth RS , Paquette D , Dostie J , Bickmore WA (2014) Spatial genome organization: contrasting views from chromosome conformation capture and fluorescence in situ hybridization. Genes Dev 28: 2778–2791 25512564 10.1101/gad.251694.114 PMC4265680

[msb20209653-bib-0166] de Wit E , de Laat W (2012) A decade of 3C technologies: insights into nuclear organization. Genes Dev 26: 11–24 22215806 10.1101/gad.179804.111 PMC3258961

[msb20209653-bib-0167] Wu Y , Shroff H (2018) Faster, sharper, and deeper: structured illumination microscopy for biological imaging. Nat Methods 15: 1011–1019 30478322 10.1038/s41592-018-0211-z

[msb20209653-bib-0168] Wutz G , Várnai C , Nagasaka K , Cisneros DA , Stocsits RR , Tang W , Schoenfelder S , Jessberger G , Muhar M , Hossain MJ *et al* (2017) Topologically associating domains and chromatin loops depend on cohesin and are regulated by CTCF, WAPL, and PDS5 proteins. EMBO J 36: 3573–3599 29217591 10.15252/embj.201798004 PMC5730888

[msb20209653-bib-0169] Xie L , Dong P , Qi Y , De Marzio M , Chen X , Banala S , Legant WR , English BP , Hansen AS , Schulmann A *et al* (2019) Super‐resolution imaging reveals 3D structure and organizing mechanism of accessible chromatin. bioRxiv 10.1101/678649 [PREPRINT]

[msb20209653-bib-0170] Xie L , Dong P , Chen X , Hsieh T‐H , Banala S , De Marzio M , English BP , Qi Y , Jung SK , Kieffer‐Kwon K‐R *et al* (2020) 3D ATAC‐PALM: super‐resolution imaging of the accessible genome. Nat Methods 17: 430–436 32203384 10.1038/s41592-020-0775-2 PMC7207063

[msb20209653-bib-0171] Xie L , Torigoe SE , Xiao J , Mai DH , Li L , Davis FP , Dong P , Marie‐Nelly H , Grimm J , Lavis L *et al* (2017) A dynamic interplay of enhancer elements regulates Klf4 expression in naïve pluripotency. Genes Dev 31: 1795–1808 28982762 10.1101/gad.303321.117 PMC5666677

[msb20209653-bib-0172] Xu CS , Hayworth KJ , Lu Z , Grob P , Hassan AM , García‐Cerdán JG , Niyogi KK , Nogales E , Weinberg RJ , Hess HF (2017) Enhanced FIB‐SEM systems for large‐volume 3D imaging. Elife 6: e25916 28500755 10.7554/eLife.25916 PMC5476429

[msb20209653-bib-0174] Yu M , Ren B (2017) The three‐dimensional organization of mammalian genomes. Annu Rev Cell Dev Biol 33: 265–289 28783961 10.1146/annurev-cellbio-100616-060531 PMC5837811

[msb20209653-bib-0175] Zheng M , Tian SZ , Capurso D , Kim M , Maurya R , Lee B , Piecuch E , Gong L , Zhu JJ , Li Z *et al* (2019) Multiplex chromatin interactions with single‐molecule precision. Nature 566: 558–562 30778195 10.1038/s41586-019-0949-1 PMC7001875

